# Surface Functionalized MXenes for Wastewater Treatment—A Comprehensive Review

**DOI:** 10.1002/gch2.202100120

**Published:** 2022-03-20

**Authors:** Lois Damptey, Bright N. Jaato, Camila Silva Ribeiro, Silvia Varagnolo, Nicholas P. Power, Vimalnath Selvaraj, David Dodoo‐Arhin, R. Vasant Kumar, Sithara Pavithran Sreenilayam, Dermot Brabazon, Vijay Kumar Thakur, Satheesh Krishnamurthy

**Affiliations:** ^1^ School of Engineering & Innovation The Open University Walton Hall Milton Keynes MK7 6AA UK; ^2^ Department of Materials Science & Metallurgy University of Cambridge 27 Charles Baggage Road Cambridge CB3 0FS UK; ^3^ School of Life Health & Chemical Sciences The Open University Walton Hall Milton Keynes MK7 6AA UK; ^4^ Department of Materials Science & Engineering University of Ghana P.O. Box LG 77 Legon‐Accra Ghana; ^5^ I‐Form Advanced Manufacturing Research Centre and Advanced Processing Technology Research Centre School of Mechanical and Manufacturing Engineering Dublin City University Glasnevin Dublin‐9 Ireland; ^6^ Biorefining and Advanced Materials Research Center SRUC Edinburgh EH9 3JG UK

**Keywords:** 2D materials, functionalization, MXene, wastewater treatment

## Abstract

Over 80% of wastewater worldwide is released into the environment without proper treatment. Whilst environmental pollution continues to intensify due to the increase in the number of polluting industries, conventional techniques employed to clean the environment are poorly effective and are expensive. MXenes are a new class of 2D materials that have received a lot of attention for an extensive range of applications due to their tuneable interlayer spacing and tailorable surface chemistry. Several MXene‐based nanomaterials with remarkable properties have been proposed, synthesized, and used in environmental remediation applications. In this work, a comprehensive review of the state‐of‐the‐art research progress on the promising potential of surface functionalized MXenes as photocatalysts, adsorbents, and membranes for wastewater treatment is presented. The sources, composition, and effects of wastewater on human health and the environment are displayed. Furthermore, the synthesis, surface functionalization, and characterization techniques of merit used in the study of MXenes are discussed, detailing the effects of a range of factors (e.g., PH, temperature, precursor, etc.) on the synthesis, surface functionalization, and performance of the resulting MXenes. Finally, the limits of MXenes and MXene‐based materials as well as their potential future research directions, especially for wastewater treatment applications are highlighted.

## Graphical abstract

2D nanomaterials, especially MXenes have recently seen exciting developments in research and development, and their application in wastewater treatment has drawn wider attention. This review examines state‐of‐the‐art strategies employed in synthesis, functionalization, and characterization of surface functionalized MXenes. Furthermore, engineering strategies like composition design, and how surface protection influence their properties as adsorbents, photocatalysts, and membranes in wastewater treatment are discussed.

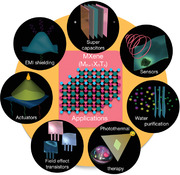

## 1 Introduction

Water is the lifeline of ecosystems, vital to human health and well‐being, and a requisite for economic prosperity.^[^ ^1^ ^]^ However, indiscriminate disposal of wastewater into global waterways is the cause of health, environmental and climate‐associated hazards.^[^ ^2^, ^3^, ^4^, ^5^, ^6^, ^7^ ^]^ The UN estimate of wastewater produced annually is approaching 1500 km^3^, almost six times more water than that exists collectively in all the rivers of the world.^[^ ^8^ ^]^ The consequences are alarming, with 4 billion cases of annually, resulting in more than 2 million deaths of children under age five.^[^ ^8^ ^]^ There has also been a significant decline in biological species in inland waters where 24% of mammals and 12% of birds connected to non‐coastal waters are considered endangered.^[^ ^8^ ^]^ The mean nitrate contaminant levels have risen ≈36% in global waterways since 1990 with the most drastic growth observed in the Eastern Mediterranean and Africa.^[^ ^5^ ^]^ Furthermore, some newer wastewater treatments have been identified to contribute remarkably to Greenhouse Gas (GHG) emissions in the form of nitrous oxide and methane—contributing threefold to the emissions of conventional wastewater treatment. In addition to these challenges, over 80% of wastewater worldwide, especially in developing countries, is released untreated into the environment.^[^ ^3^ ^]^

To address these issues, conventional techniques like chemical precipitation, membrane filtration, solvent extraction, ion exchange, electrochemical removal, and coagulation have been utilized and continue to be used as water treatment techniques.^[^ ^9^ ^]^ The challenges associated with these methods are incomplete removal of impurities, high energy inefficiency, production of toxic sludge, inefficient and sensitive operating methods.^[^ ^10^ ^]^ These bottlenecks have, however, paved the way for the heightened interest in the unique properties of 2D nanomaterials within the scientific community, of which MXenes have been identified as a promising aspirant for wastewater treatment.^[^ ^11^ ^]^

**Figure 1:**
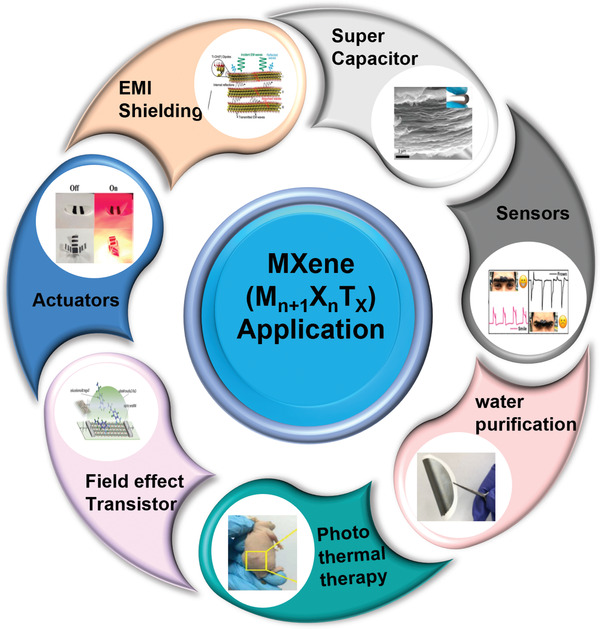
MXene Applications: actuators (Reproduced with permission.^[^ ^20^ ^]^ Copyright 2019, Springer Nature), EMI shielding (Reproduced with permission.^[^ ^21^ ^]^ Copyright 2019, Elsevier), supercapacitor (Reproduced with permission.^[^ ^22^ ^]^ Copyright 2017, Springer Nature), sensors (Reproduced with permission.^[^ ^23^ ^]^ Copyright 2017, RSC), water purification (Reproduced with permission.^[^ ^17^ ^]^ Copyright 2020, Wiley‐VCH), photothermal therapy (Reproduced with permission.^[^ ^24^ ^]^ Copyright 2013, American Ceramic Society) and field effect transistor (Reproduced with permission.^[^ ^25^ ^]^ Copyright 2019, Elsevier).

MXenes are a relatively new and rapidly developing family of 2D materials with general formula M*_n_*_+1_X*_n_* T*_x_* and are derived from the precursor MAX phase (M*_n_*_+1_AX*_n_*), where the integer *n* is 1, 2, or 3, M is an early transition metal, X is either carbon, nitrogen or both, A represents a group IIIA or IVA element, and T represents surface terminal groups like fluorine, oxygen, chlorine, or hydroxyl, and *x* denotes the number of the surface functional group.^[^ ^11^, ^15^, ^17^, ^18^, ^19^, ^20^ ^]^ The incredible intrinsic features of MXenes such as high surface area, high metallic conductivity, ease of functionalization, environmentally friendly nature, antibacterial properties, chemical stability, activated metallic hydroxide sites, and hydrophilicity, make them the preferred candidates for applications in environmental remediation, energy storage, catalysis, sensors, and electronics (**Figure** **1**).^[^ ^11^, ^12^, ^13^, ^16^, ^18^, ^21^, ^22^, ^23^, ^24^, ^25^, ^26^ ^]^ One of the major advantages that MXenes have over other 2D materials is in the ease of tuning their chemical and physical properties by simply changing the MAX phase precursor or modifying the surface functional groups.^[^ ^22^ ^]^ These processes are known to alter the energy storage capacity, modify the magnetic properties, optimize band gap, change the surface plasmon resonance and modify the electron transport properties of MXenes. Consequently, the T groups in MXenes have gained significant experimental and theoretical attention.^[^ ^22^ ^]^ The presence of abundant oxygen‐containing terminal groups on the surface of MXenes provides advantageous active sites for chemical covalent modification of the surface. This makes it possible to engineer new properties by introducing appropriate functional groups, thus enhancing the performance of MXenes for removing pollutants from wastewater.

It is worth noting that a few reviews are available covering MXene and MXene‐based material for environmental applications.^[^ ^27^, ^28^, ^29^ ^]^ Cases in point Jun et al. presented a detailed review on MXene nanomaterial in energy conversion and storage, adsorption, membranes, photocatalysis, and antimicrobial applications,^[^ ^30^ ^]^ whereas Chen et al. presented a minireview focusing on surface modification of MXenes, their application in environmental adsorption and catalytic degradation, their biocompatibility, and toxicity.^[^ ^31^ ^]^ In another review, Rasool presented the synthesis and application of MXenes for application as adsorbents, desalination membranes, electrodes for electrochemical deionization, and as antibacterial agents for water purification and other environmental applications.^[^ ^32^ ^]^ Al‐Hamadani focused on MXene‐based membranes and the effect of several factors on liquid separation during membrane filtration,^[^ ^14^ ^]^ Zhang presented an interesting minireview on the adsorptive remediation of environmental pollutants using MXenes,^[^ ^33^ ^]^ Jeon explored the various effects of water qualities on the adsorptive properties of MXene‐based adsorbents,^[^ ^34^ ^]^ Ihsanullah emphasized the adsorption‐reduction properties of MXenes and MXene‐based materials in water treatment applications,^[^ ^35^ ^]^ and also critically reviewed the synthesis and application of MXenes in desalination,^[^ ^18^ ^]^ Ibrahim reviewed the synthesis, characterization techniques, advantages and drawbacks of MXene‐based materials for adsorption of toxic metals,^[^ ^36^ ^]^ Kumar comprehensively reviewed the structural patterns, synthesis, properties and application of MXenes for the removal of pollutants like radionuclides and dyes from water, as well as the mechanism of pollutant removal,^[^ ^37^ ^]^ Khatami takes an environmental perspective and analyses the environmental risks associated with the deployment MXenes and MXene‐based materials in wastewater treatment,^[^ ^38^ ^]^ Zhong reviewed the various environmental photocatalytic applications of MXene and MXene‐based materials focusing especially on the synthesis techniques,^[^ ^39^ ^]^ Feng surveyed the photocatalytic applications of MXene and MXene‐based materials in degrading pollutants in water, discussed the surface properties, and improvement in performances realized by introducing heterojunctions and Schottky junctions,^[^ ^40^ ^]^ Huang presented a review on surface modified MXenes and their biomedical application,^[^ ^41^ ^]^ Zhan reviews the synthesis, properties and application of MXene and MXene‐based composites in various environmental‐related applications,^[^ ^42^ ^]^ Yu gives a review on the synthesis and properties, and removal mechanism of pollutants and the related toxicity of MXenes and MXene‐based materials in water treatment,^[^ ^43^ ^]^ Javaid reviews MXene‐based hybrid materials with special focus on their photocatalytic application in the elimination of pharmaceuticals from wastewater,^[^ ^44^ ^]^ Sun et al. critically reviewed and summarized the synthesis and properties of MXenes to demonstrate the key roles in ameliorating their adsorption performance as shown in the removal of gases, organics, heavy metals, and radionuclides.^[^ ^45^ ^]^ In more recent publications, Kwon gives a general review on the synthesis, surface chemistry, interlayer tuning, membrane fabrication, and application of MXene‐based materials for water purification, Dixit presents a state‐of‐the‐art review on water treatment and desalination using MXene composites,^[^ ^46^ ^]^ Berkani reviews the fabrication methods, structural and chemical modifications, and application of MXene and MXene‐based composites for water treatment,^[^ ^47^ ^]^ Sheth reviews synthesis techniques, optimization of desired adsorption properties, regeneration and adsorption mechanism of MXenes in removing noxious pollutant from water,^[^ ^48^ ^]^ and Vasseghian reviews the sonocatalytic degradation of pollutants using MXene‐based catalysts.

In this work, we present a comprehensive review of state‐of‐the‐art developments in the synthesis, characterization, and surface functionalization of MXenes and MXene‐based materials, for wastewater treatment. First, we introduce the sources and types of pollutants present in wastewater, the consequent health and environmental effects of these pollutants, and the conventional remediation techniques employed in wastewater treatment. This is then followed by a description of MXenes as a class of compounds, their (surface) chemistry, synthesis and surface functionalization routes, characterization techniques, and their application (as adsorbents, photocatalysts, antifouling/antibacterial agents, and membranes) in the removal of toxic pollutants (dyes, heavy metals, pharmaceutical wastes, bacteria, and radioactive wastes) from wastewater. We also discuss how a range of factors (e.g., PH, temperature, precursor, etc.) affect the synthesis, surface functionalization, and performance of the resultant MXene‐based material. Finally, we highlight the limits of MXenes and MXene‐based materials as well as their potential future research directions, especially for wastewater treatment applications. We believe this review will attract the attention of scientists in material, chemistry, and related fields and promote the development of MXene and MXene‐based materials to address the global challenge of wastewater treatment and water scarcity.

## 2 Wastewater: Sources, Composition and Effects

**Figure 2:**
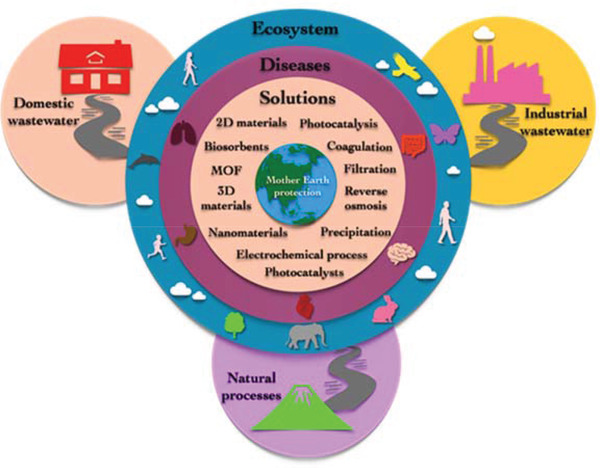
Domestic, industrial, and natural sources of wastewater.

Wastewater can be defined as polluted water generated from homes, communities, farms, or industries that contain dissolved or suspended matter. Wastewater is made up of ≈0.06% solids dissolved or suspended in 99.94% water flow.^[^ ^49^ ^]^ Depending on the source of the solid loads, the resulting wastewater is classified into industrial, domestic, and storm sewage (natural processes) wastewater as depicted in **Figure** **2**.^[^ ^49^ ^]^ Water is indispensable and serves various purposes in many industries including, petroleum,^[^ ^50^, ^51^ ^]^ mining,^[^ ^52^ ^]^ pharmaceutical,^[^ ^53^, ^54^ ^]^ textile,^[^ ^55^ ^]^ fertilizer^[^ ^52^ ^]^ pulp and paper^[^ ^53^ ^]^ and pesticides,^[^ ^56^ ^]^ and a substantial proportion of this water ends up as industrial wastewater. For example, manufacturing or chemical processes and their resultant discharge with the associated pollutants (e.g., heavy metal, aromatic hydrocarbons and alkyl phenols, naturally occurring radioactive material, cyanides, ammonia, organochlorine‐based pesticides, pigments, dyes, arsenic trioxide) released into the environment creates significant and detrimental footprints and related health hazards.^[^ ^57^ ^]^ Domestic wastewater from homes is a primary source of microorganisms (e.g., pathogens, virus, eggs of worms and Protista,^[^ ^55^, ^58^ ^]^ putrescible organic materials (e.g., proteins, carbohydrates, and fat),^[^ ^55^, ^58^ ^]^ metals (e.g., lead (Pb), cadmium (Cd), chromium (Cr), copper (Cu), nickel (Ni), and mercury (Hg),^[^ ^55^, ^58^ ^]^ and plant nutrients (e.g., nitrogen, phosphorus, ammonium).^[^ ^55^, ^58^ ^]^ These pollutants present a direct threat to public health and water quality. Furthermore, pharmaceuticals such as ibuprofen, metoprolol, paracetamol, and sulfamethoxazole, when released into the environment can be highly toxic to humans, animals, and aquatic life.^[^ ^50^, ^53^, ^59^ ^]^ Storm sewage includes sewage overflow and road runoff from precipitation that is collected in a system of pipes or open channels and may contain nitrogen and phosphorus pollutants from fertilizers, yard, and petrochemical wastes that pollute water bodies during heavy rainfall or snowmelt.^[^ ^60^ ^]^

The main objectives for wastewater treatment are to reduce the levels of solids, biodegradable organic matter, pathogens, and other toxic compounds in wastewater, to meet regulatory limits that are protective of both public health and the environment.^[^ ^59^, ^61^ ^]^ The contamination of wastewater is generally given in terms of chemical oxygen demand (COD), biochemical oxygen demand (BOD), dissolved oxygen (DO), suspended solids, and total dissolved solids (TDS).^[^ ^62^ ^]^ To achieve the above objectives, wastewater treatment systems combine physical, chemical, and biological approaches to get rid of pollutants in wastewater.

**Figure 3:**
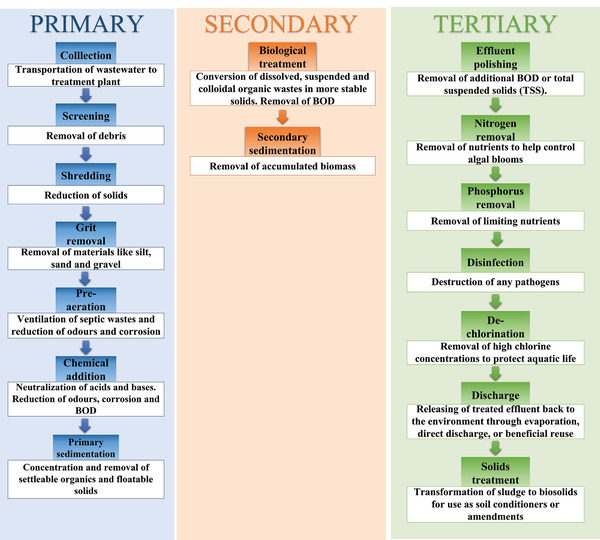
Classification and processes involved in conventional wastewater treatment.^[^ ^49^, ^53^, ^59^ ^]^

Wastewater treatment plants combine physical, chemical, and/or biological methods to provide three levels of treatment: primary, secondary, and tertiary treatments (**Figure** **3**). Primary treatment involves physical techniques for the removal of suspended solids by sedimentation, screening, and filtration, typically eliminating up to 35% of the BOD and 60% of the suspended solids.^[^ ^60^, ^63^, ^64^, ^65^ ^]^ A variety of chemical techniques are also harnessed to precipitate, convert, or destroy contaminants, for example, solids are removed by coagulation and flocculation, pathogens are destroyed by disinfection, and nutrients like phosphorus are eliminated by chemical precipitation. Finally, biological techniques help to remove biodegradable organic matter and non‐settleable colloidal solids using microorganisms.^[^ ^60^, ^63^, ^64^, ^65^ ^]^ Secondary treatment using physical and biological methods remove ≈90% of the BOD and 90% of the suspended solids.^[^ ^60^, ^66^ ^]^ Pollutants that escape removal via the secondary treatment process undergo tertiary treatment typically involving the removal of nitrogen, phosphorus, soluble COD, and heavy metals.^[^ ^64^, ^65^ ^]^

In more recent years, nanomaterials have come under intense research and development, and their application in water and wastewater treatment has drawn wider attention.^[^ ^67^, ^68^ ^]^ In particular, 2D nanomaterials are suitable alternatives for existing water and wastewater purification methods.^[^ ^14^, ^67^, ^68^, ^69^, ^70^, ^71^, ^72^, ^73^, ^74^, ^75^ ^]^

### 2.1 2D Materials for Wastewater Treatment

Since the discovery of the single‐atomic thick graphene in 2004, and the revelation of its excellent electron transport capacity, electrical conductivity, thermal conductivity, mechanical characteristics, and chemical properties,^[^ ^72^, ^73^ ^]^ a diverse range of other 2D nanomaterials have been explored and subjected to intensive and extensive studies in the fields of condensed matter physics, chemistry, material science, and nanotechnology.^[^ ^73^ ^]^ 2D materials, which are sheet‐like structures with single‐ or few‐layer thickness (<5 nm), and lateral sizes that range beyond 100 nm,^[^ ^74^, ^75^, ^76^ ^]^ are promising candidates for wastewater treatment and other environmental remediation applications.^[^ ^32^, ^74^ ^]^

The properties of 2D materials are dictated to a greater extent by how they are prepared, and the techniques employed in their preparation can be grouped into either a top‐down or bottom‐up approach.^[^ ^77^, ^78^, ^79^, ^80^ ^]^ The top‐down approach includes mechanical cleavage and solution‐based exfoliation, which works to overcome the Van der Waals energy stored in the bulk crystals or parent layered material by exerting an external force such as peeling or ultrasonication. Conversely, the bottom‐up approach includes chemical vapor deposition (CVD) and wet chemical synthesis, where precursors or smaller molecules are chemically reacted to synthesize layered materials on substrates or in solution.^[^ ^77^, ^78^, ^79^, ^80^, ^81^ ^]^ MXenes as a class of 2D materials are prepared via the top‐down approach and the process involves chemical etching and subsequent rupturing of Van der Waals forces. In the next section, attention is focused on the chemistry, synthesis, characterization, and applications of MXenes and their surface‐modified form.

## 3 MXenes

**Figure 4:**
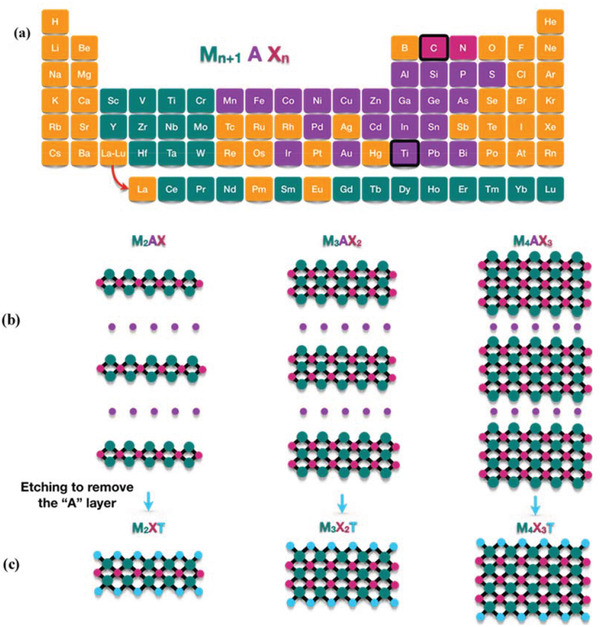
a) MXene composition: M is an early transition metal (green), A = group IIIA or IVA elements (purple), X is carbon/nitrogen or both (red). b) Structure of MAX phases and c) the corresponding MXenes.

MXenes represent a family of 2D early transition‐metal (M) carbides and nitrides, which were first synthesized from bulk layered crystalline M*_n_*_+1_AX*_n_* (*n* = 1, 2, 3),^[^ ^31^, ^82^, ^83^, ^84^ ^]^ where M is an early transition metal, A = group IIIA or IVA elements, X is carbon/nitrogen or both. By etching out the A element as shown in **Figure** **4**a, MXenes with the general formula M*_n_*_+1_ X*_n_* T*_x_* (*n* = 1, 2, 3) are obtained, where T stands for the surface terminal groups (—OH, —F, ‐O—) that render MXenes hydrophilic (*x* denotes their number).^[^ ^85^, ^86^ ^]^ The first example of MXene material, Ti_3_C_2_, was synthesized by selectively etching the Al atoms in the layered hexagonal ternary carbide, Ti_3_AlC_2_, using hydrofluoric acid (HF) at room temperature.^[^ ^87^ ^]^ Figure 4b is a schematic showing the structures of MX_2_, M_3_X_2_, and M_4_X_3_.

### 3.1 Synthesis and Chemistry of MXenes

Typically, MXene is prepared from the parent MAX phase. The MAX phase has three types of unit cells with hexagonal crystal structure (space group *P63/mmc*) with metallic, covalent, and ionic bonds.^[^ ^88^, ^89^ ^]^ The A‐group layer is relatively weak and more reactive and is between closely packed M layers, and X atoms (occupying the octahedral sites).^[^ ^90^ ^]^ The bond between M and X is exceptionally strong exhibiting a mixed covalent/metallic/ionic characteristic such that they are inseparable by mechanical exfoliation, ultrasonication, or dispersion.^[^ ^90^, ^91^ ^]^ The bonding mode of MXene is different from those of other layered materials like graphite in which weak van der Waals interactions hold similar structures together.^[^ ^90^ ^]^ When the A element is etched out, the exposed “M” becomes very reactive and easily bonds with electronegative atoms (like F, O, or OH resulting in the T*_x_* group shown in the formula M*_n_*_+1_ X*_n_* T*_x_*.^[^ ^82^, ^90^ ^]^ Experimentally, the surface termination groups present in the MXene depend entirely on the synthesis conditions (such as etchant used, etching and delamination conditions, the types of M element, post synthetic procedure, and storage) employed during synthesis.^[^ ^92^, ^93^ ^]^ It is these surface functional groups that dictate the properties of the resulting MXenes. For example, studies by Agresti predict that bandgap energies of MXenes could range from 1.67 to 6.25 eV when OH and O surface terminal groups, respectively.^[^ ^94^ ^]^ This suggests that by introducing and optimizing surface terminal groups, the bandgap energy, and work function properties can be tuned. The easy functionalization of MXenes makes them very versatile in various applications, as new properties can be easily conferred on them.

The pioneering on MXenes by Naguib et al. reported the synthesis by immersing Ti_3_AlC_2_ powders in 50 wt% HF solution for 2 h at room temperature, followed by washing with deionized and centrifugation to precipitate the powder.^[^ ^82^, ^95^ ^]^ The proposed mechanism for this reaction was the selective etching of the Al layers from Ti_3_AlC_2_ (Reaction (1)) and summarized below.^[^ ^96^ ^]^

(1) $$\begin{array}{c} {\text{Ti}}_{3}AlC_{2}+3HF\to AlF_{3}+1.5H_{2}+{\text{Ti}}_{3}C_{2} \end{array}$$

(2) $$\begin{array}{c} {\text{Ti}}_{3}C_{2}+2H_{2}O\to {\text{Ti}}_{3}C_{2}{\left(OH\right)}_{2}+H_{2} \end{array}$$

(3) $$\begin{array}{c} {\text{Ti}}_{3}C_{2}+2HF\to {\text{Ti}}_{3}C_{2}F_{2}+H_{2} \end{array}$$

The formation of surface groups (OH and F) on the exfoliated 2D Ti_3_C_2_ layers is depicted by Reactions (2) and (3).^[^ ^97^, ^98^ ^]^

### 3.2 Surface Termination

As mentioned previously, etching of the A layer from the MAX phase often leaves —O, —OH, and/or —F functional groups on the surface of MXenes (M*_n_*_+1_ X*_n_* (OH)*_x_* O*_y_* F*_z_*).^[^ ^99^ ^]^ These electronegative terminal groups allow for easy surface modification with organic groups and have been reported to significantly regulate the physical and chemical properties of MXenes. It has been shown that 2D titanium carbide possesses unique properties from a decrease in F surface groups and an increase in OH groups.^[^ ^100^ ^]^

### 3.3 MXene Characterization

Evidence of successful surface functionalization of MXenes has been studied using various material characterization techniques. These techniques reveal information about the chemical composition and crystal structures before and after surface modification MXene. Knowledge about the composition and distribution of surface functional groups —O, —OH, and —F has been explored in different applications. Furthermore, the morphology and interlayer distance of the MXene flakes before and after surface functionalization have also been studied.^[^ ^17^ ^]^ The techniques employed for this purpose include X‐Ray diffractometer (XRD),^[^ ^85^, ^87^, ^101^ ^]^ X‐Ray photoelectron spectroscopy (XPS),^[^ ^86^, ^102^ ^]^ scanning electron microscopy (SEM), transmission electron microscopy/high‐resolution transmission electron microscopy (TEM/HRTEM),^[^ ^103^, ^104^, ^105^, ^106^ ^]^ energy dispersive X‐ray analysis (EDAX),^[^ ^107^, ^108^ ^]^ Fourier transform infrared spectroscopy (FTIR),^[^ ^109^, ^110^ ^]^ Brunauer–Emmett–Teller analysis (BET), Raman spectroscopy,^[^ ^111^, ^112^, ^113^, ^114^ ^]^ and thermogravimetric analysis (TGA).^[^ ^101^, ^112^, ^115^, ^116^ ^]^ MXenes have been functionalized with several materials ranging from metal nanoparticles to cellulose aerogel, amino groups, etc. to enhance their properties and performance toward wastewater treatment.^[^ ^111^, ^117^, ^118^, ^119^, ^120^ ^]^

#### 3.3.1 X‐Ray Diffraction

**Figure 5:**
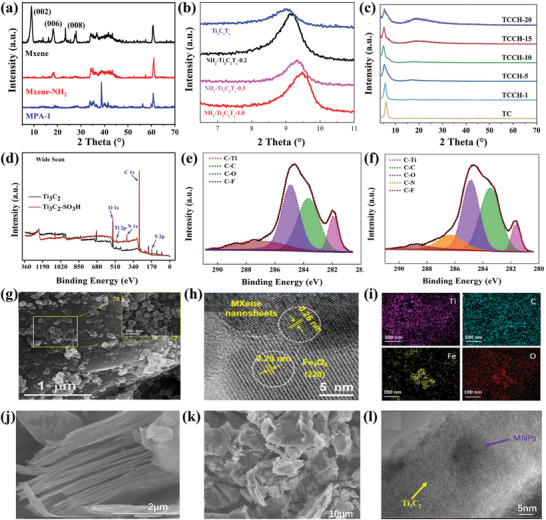
XRD patterns of a) Ti_3_C_2_ MXene, amino functionalized MXene (MXene‐NH_2_), and MXene/Polyethylenimine/Sodium alginate (labeled as MPA) showing disappearance of the (0002) peak and reduction in the intensity of the (006) and (008) peak. Reproduced with permission.^[^ ^117^ ^]^ Copyright 2021, Elsevier. b)Ti_3_C_2_T*_x_*, NH_2_‐Ti_3_C_2_T*_x_*‐0.2, NH_2_‐Ti_3_C_2_T*_x_*‐0.5 and NH_2_‐Ti_3_C_2_T*_x_*‐1.0 showing shift in (002) from 9.02° (Ti_3_C_2_T*_x_*) to 9.20° (NH_2_‐Ti_3_C_2_T*_x_*‐0.2), 9.32° (NH_2_‐Ti_3_C_2_T*_x_*‐0.5) and 9.48°(NH_2_‐Ti_3_C_2_T*_x_*‐1.0). Reproduced with permission.^[^ ^118^ ^]^ Copyright 2021, Elsevier. c) XRD of a series of carboxyl modified MXene with different reaction ratios of diazonium salt. Reproduced with permission.^[^ ^139^ ^]^ Copyright 2020, Elsevier. XPS wide scan spectrum of d) pristine Ti_3_C_2_ and Ti_3_C_2_–SO_3_H and narrow scan spectra of Ti_3_C_2_–SO_3_H showing the C 1s, N 1s, O 1s, S 2p, Ti 2p peaks. Reproduced with permission.^[^ ^127^ ^]^ Copyright 2019, Elsevier. Multilayer Ti_3_C_2_T*_x_* e) and NH_2_‐Ti_3_C_2_T*_x_* f) rising of a new peak of C1s which occurs at 286.3 eV which was assigned to C—N. Reproduced with permission.^[^ ^118^ ^]^ Copyright 2021, Elsevier. g) SEM image of MXene@Fe_3_O_4_ demonstrating the 2D lamellar structure with spherical shape Fe_3_O_4_ nanoparticles (inset), h) High‐resolution TEM (HRTEM) and i) elemental mapping analyses (Ti: purple, C: green, Fe: yellow, and O: red) of MXene@Fe_3_O_4_. Reproduced with permission.^[^ ^137^ ^]^ Copyright 2019, American Chemical Society. SEM images of j) pristine Ti_3_C_2_ MXene and k) MX‐MNPs, l) HRTEM of MX‐MNPs. Reproduced with permission.^[^ ^137^ ^]^ Copyright 2020, Elsevier.

The crystal structure and phase arrangement of as prepared and surface modified MXene have been explored employing X‐ray diffraction (XRD) characterization.^[^ ^28^, ^107^, ^112^, ^121^, ^122^ ^]^ Pristine MXene usually shows a characteristic peak of the (002) plane at ≈7.51° with 1.18 nm spacing between the layers.^[^ ^113^, ^123^, ^124^ ^]^ Feng et al. fabricated MXene/PEI/SA (MPA) composite aerogel by first functionalizing pristine MXene with (3‐aminopropyl)trimethoxysilane (APTES) to obtain an amino‐functionalized MXene (MXene‐NH_2_). The amino‐functionalized product was then transformed into MPA using polyethylenimine (PEI) (which served functional components like MXene‐NH_2_), sodium alginate (SA) (as an aerogel carrier), and epichlorohydrin (as a crosslinking agent). When the XRD data of MXene‐NH_2_ was compared to that of pristine MXene, they observed reduced peak intensities at 2θ = 18.2 at 2θ = 27.84° for the (006) and (008) peaks, respectively, and disappearance of thee (002) peak at 2θ = 8.86° (**Figure** **5**a).^[^ ^117^ ^]^ The peak intensities of the (006) and (008) peaks further diminished when MPA was formed (Figure 5a). They attributed this observation to the formation of a 3D architecture with strong chemical, electrostatic, and hydrogen interactions that enhanced the stability of the composite, subsequently resulting in its high adsorptive feature for Cr (VI).^[^ ^117^ ^]^ In another study, Kong and his colleagues functionalized MXene with varying quantities of the electron rich APTES for Cr(VI) adsorption and observed as is shown in Figure 5b that the (002) peak shifted toward relatively high 2θ values from 9.02° for Ti_3_C_2_T*_x_* to 9.20° (NH_2_‐Ti_3_C_2_T*_x_*‐0.2), 9.32° (NH_2_‐Ti_3_C_2_T*_x_*‐0.5) and 9.48° (NH_2_‐Ti_3_C_2_T*_x_*‐1.0).^[^ ^118^ ^]^ This depicts tightly packed NH_2_‐Ti_3_C_2_T*_x_* nanosheets via favorable electrostatic or hydrogen interactions between the separated ionic layers.^[^ ^118^, ^125^, ^126^ ^]^ Lei et al., after modifying Ti_3_C_2_ MXene with sulfonic acid to form Ti_3_C_2_‐SO_3_H, observed a downward shift in the peak position of the (002) phase of pristine Ti_3_C_2_ from a 2θ value of 9.52° to 6.16°. Besides, the (002) peak became sharper for the Ti_3_C_2_‐SO_3_H because of the thickening of the MXene layer.^[^ ^124^, ^127^ ^]^ The XRD technique was employed by Jiang et al., to show that the 002 peak of MXene, which they observed at 7°, was well preserved even after functionalizing with a diazonium salt. This indicates that the covalent grafting of the salt does not result in the collapse of the layered structures of the MXene nanosheets (Figure 5c).^[^ ^128^ ^]^

#### 3.3.2 XPS

XPS is a widely known surface analysis technique employed X to determine the oxidation states and chemical environment of materials during characterization.^[^ ^129^, ^130^, ^131^, ^132^ ^]^ In surface‐functionalized MXene studies, this technique has been employed to analyze the surface chemical states and composition of prepared materials. For pristine Ti_3_C_2_T*_x_* (the most widely used form of MXene), the elements that exist are usually Ti 2p at 463.64 eV, C 1s at 284.8 eV, and O *s* at 532.0 eV. Depending on the precursor used during functionalization, evidence of its elements will be observed on the surface of the prepared sample. A typical example of an XPS survey scan for Ti_3_C_2_ MXene functionalized with the sulfonic group (SO_3_H) shows the presence of sulfur 2p which can be resolved into 2 peaks at 168.85 eV and 167.9 eV (Figure 5d). The presence of S 2p confirms the successful functionalization of the Ti_3_C_2_ MXene.^[^ ^133^ ^]^ By means of XPS, Kong et al., after modifying MXene with APTES, observed new peaks of C 1s and O 1s appearing at 286.3 and 531.7 eV for the newly formed C‐N and Ti‐O‐Si bonds, respectively (Figure 5f).^[^ ^134^ ^]^

#### 3.3.3 SEM/TEM/Energy Dispersive Spectroscopy

Surface morphology, microstructure, and specific elemental composition of functionalized MXenes nanocomposites are characterized with the SEM and TEM or High‐Resolution TEM (HRTEM) usually coupled with energy dispersive spectroscopy /energy dispersive X‐ray analysis (EDS/EDAX). Pristine Ti_3_C_2_T*_x_* MXene exhibits multilayer 2D morphology (Figure 5j) with packed lamellar layer structures.^[^ ^135^ ^]^

Zhang et al. prepared magnetic MXene nanocomposite functionalized with Fe_3_O_4_ (MXene@ Fe_3_O_4_). A 2D lamellar structure morphology was observed for MXene@ Fe_3_O_4_ with spherical Fe_3_O_4_ (with an average diameter of ≈55 nm) uniformly covering the entire MXene surface (Figure 5g). HRTEM confirmed a lattice spacing of 0.29 nm corresponds to the (220) d spacing of the crystalline Fe_3_O_4_, and the 0.26 nm for the Ti_3_C_2_ facet (Figure 5h). Elemental mapping from EDS indicated the presence and distribution of Ti, C, Fe, O, and N in the composite (Figure 5i).^[^ ^136^ ^]^ In a similar vein, Cui et al. also prepared composites of Ti_3_C_2_ MXene and Fe_3_O_4_ magnetic nanoparticles (MNPs), which they labeled MX‐MNPs. The prepared MX‐MNPs were observed to possess a different morphology (Figure 5k) from that of pristine MXene (Figure 5j), with MNPs well dispersed on the surface (Figure 5l).^[^ ^120^, ^137^ ^]^

In another study, Ti_3_C_2_T*_x_* was functionalized with l‐3,4‐dihydroxyphenylalanine (DOPA) to form Ti_3_C_2_T*_x_*‐PDOPA. The Ti_3_C_2_T*_x_*‐PDOPA composite was found to be more transparent than the starting Ti_3_AlC_2_ material, which is an important feature in photocatalysis for water treatment. TEM revealed that small aggregates of PDOPA were evenly distributed on the surface of Ti_3_C_2_T*_x_*.^[^ ^123^ ^]^ In their recent publication on the synthesis of ternary g‐C_3_N_4_/TiO_2_/Ti_3_C_2_ MXene photocatalyst, Hu et al. showed via SEM images that TiO_2_‐Ti_3_C_2_ MXene calcined at 450 °C were nanosheets and graphitic carbonitride (g‐C_3_N_4_) were a micro‐sized particle.^[^ ^138^ ^]^ They used EDS mapping to confirm the presence of Ti, O, C, and N. HRTEM revealed the lattice spacing of 0.35 nm which corresponds to (101) planes of anatase TiO_2_. An amorphous phase of CN was observed to provide a heterogeneous structure that serves as a photocatalytic active center and an effective transfer path for photoinduced carriers.^[^ ^138^ ^]^

#### 3.3.4 BET

**Figure 6:**
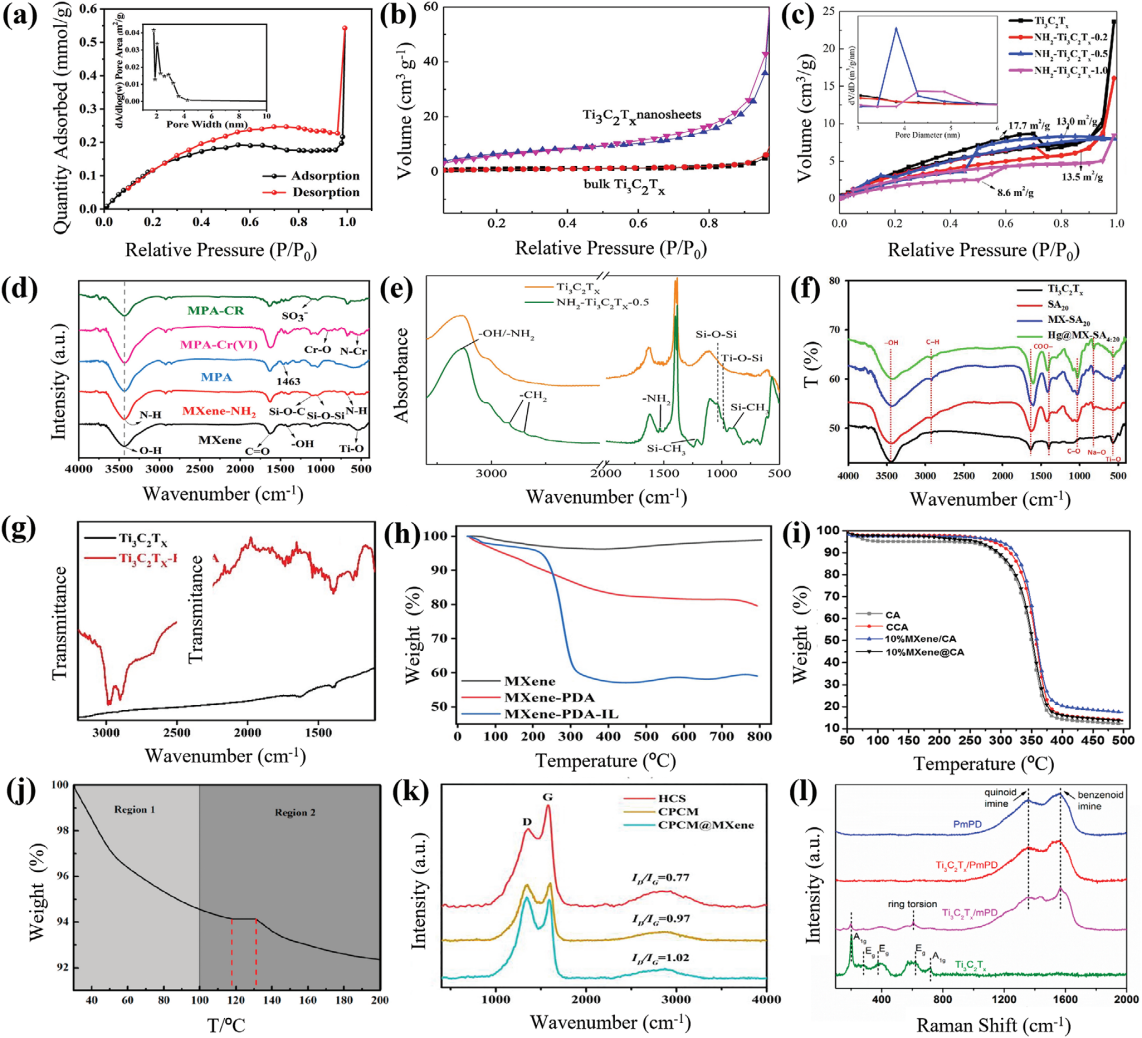
a) N_2_ adsorption‐desorption isotherms of MXene/PEI modified sodium alginate aerogel (MPA) exhibiting a typical Type‐IV curve with H3 hysteresis (inset is the corresponding pore size distribution of MPA). Reproduced with permission.^[^ ^117^ ^]^ Copyright 2021, Elsevier. b) N_2_ adsorption‐desorption isotherms of bulk Ti_3_C_2_T*_X_*_ _and the Ti_3_C_2_T*_X_* nanosheets obtained by the expansion method. Reproduced with permission.^[^ ^102^ ^]^ Copyight 2021, Elsevier. c) Specific surface area of Ti_3_C_2_T*_X_* and NH_2_‐Ti_3_C_2_T*_x_* with different contents of APTES (inset shows the mesopores size distribution). Reproduced with permission.^[^ ^118^ ^]^ Copyright 2021, Elsevier. d) FT‐IR spectra of MXene, MXene‐NH_2_, MPA, MPA‐Cr(VI) and MPA‐CR. Reproduced with permission.^[^ ^117^ ^]^ Copyright 2021, Elsevier. e) FT‐IR spectra of Ti_3_C_2_T*_X_* and NH_2_‐ Ti_3_C_2_T*_X_*‐0.5 nanosheets. Reproduced with permission.^[^ ^118^ ^]^ Copyright 2021, Elsevier. f) FT‐IR of Ti_3_C_2_T*_X_* MXene, SA20, MX‐SA_4:20_, and Hg^2+^@MX SA_4:20_. Reproduced with permission.^[^ ^147^ ^]^ Copyright 2019, Elsevier. g) FT‐IR spectra of MXene and MXenes with PDOPA. Reproduced with permission.^[^ ^123^ ^]^ Copyright 2019, Elsevier. h) TGA curves of MXene, MXene‐PDA (polydopamine), and MXene‐PDA‐IL (from room temperature to 800 °C under N2 ambient). Reproduced with permission.^[^ ^101^ ^]^ Copyright 2021, Elsevier. i) Thermogravimetric analysis of CA (cellulose acetate), CCA (cross‐linked CA), 10%MXene/CA, and 10%MXene@CA membranes.^[^ ^112^ ^]^ j) TGA of MXene‐carbon nanotube membranes with two stages of weight loss from 30 to 200 °C. Reproduced with permission.^[^ ^116^ ^]^ Copyright 2021, Elsevier. k) Raman spectra showing the characteristic signals of the HCS (hydrothermal carbon sphere), CPCM, and MXene/chitosan‐based porous carbon microspheres (CPCM@MXene) at 1350 cm^−1^ (D band) and 1590 cm^−1^ (G band). Reproduced with permission.^[^ ^85^ ^]^ Copyright 2021, Elsevier. l) Raman spectra of Ti_3_C_2_T*_x_*, PmPD (poly(m‐phenylenediamine), and Ti_3_C_2_T*_x_*/PmPD. Reproduced with permission.^[^ ^111^ ^]^ Copyright 2019, Environmental Research and Public Health.

BET has been employed to determine the specific surface area, average pore diameter, and total pore volume of pristine MXene and their functionalized forms.^[^ ^140^, ^141^ ^]^ BET works through gas physical adsorption on the surface of a material by calculating the mass of adsorbed gas corresponding to a monomolecular layer on a particle surface. The physical adsorption results measured are from weak Van der Waal forces on the surface of materials and the adsorbed nitrogen gas molecules.^[^ ^142^ ^]^ Feng et al., after preparing MXene/PEI/SA (a hierarchical porous structure with mesopores between cross linked SA and PEI with the Ti_3_C_2_T*_x_* MXene), established by BET analysis its specific surface area to be 16.31 m^2^ g^−1^ with a pore size diameter of 2–5 nm (**Figure** **6**a). Microporous structures were formed by ice crystal growth during freeze drying. They concluded that the presence of these structures promoted the adsorption of Cr(VI) and Congo red, thus the increased performance of MXene/PEI/SA nanostructure was attributed to the availability of macropores for adsorption of larger molecules such as dyes, whiles mesopores enhanced capillary effect.^[^ ^117^ ^]^

Zhao et al. also reported that after functionalizing the surface of Cu_2_O with different weight percentages of Ti_3_C_2_T*_x_* MXene (1% – 10%), there was a considerable increase in the specific surface area of pristine Cu_2_O from 16.24 to 27.25 m^2^ g^−1^ with an increase in BET surface area of Ti_3_C_2_T*_X_*‐nanosheets to about seven times higher than that of bulk Ti_3_C_2_T*_x_* (Figure 6b).^[^ ^102^ ^]^ The 10% Ti_3_C_2_T*_x_* nanosheets/Cu_2_O sample showed the highest surface area (**Table** **1**) which contributed to the highest adsorption of tetracycline (TC) hence enhancing its photocatalytic removal efficiency. In another example, different proportions of APTES were covalently cross linked onto the surface of Ti_3_C_2_T*_x_* MXene to produce NH_2_‐Ti_3_C_2_T*_x_* (denoted as NH_2_‐Ti_3_C_2_T*_x_*‐0.2, NH_2_‐Ti_3_C_2_T*_x_*‐0.5, and NH_2_‐Ti_3_C_2_T*_x_*‐1, respectively). It was observed that NH_2_‐Ti_3_C_2_T*_x_*‐0.5 nanosheet possessed the highest adsorption performance (93.0 mg g^−1^) for Cr(VI) due to the positively charged surface and its specific surface area of 13.2 m^2^ g^−1^ as compared to pristine Ti_3_C_2_T*_x_* (52.2 mg g^−1^) with surface areas of 17.7 m^2^ g^−1^ (Figure 6c).^[^ ^118^ ^]^ A high surface area is an important characteristic for various fields such as catalysis.^[^ ^130^ ^]^

**Table 1:** Results obtained from BET analysis on various surface functionalized MXenes

Ref.	Surface functionalized MXene	Specific surface area [m^2^ g^−1^]	Pore size distance [nm]
^[^ ^117^ ^]^	Ti_3_C_2_T*_x_* MXene/PEI/sodium alginate	16.31	2–5
^[^ ^118^ ^]^	Ti_3_C_2_T*_x_*	17.7	3.05
	NH_2_‐Ti_3_C_2_T*_x_* – 0.2	13.5	3.05
	NH_2_‐Ti_3_C_2_T*_x_*_–_ 0.5	13.0	3.82
	NH_2_‐Ti_3_C_2_T*_x_*‐1	8.6	4.29
^[^ ^110^ ^]^	Fe_3_O_4_@Ti_3_C_2_T*_x_* ‐BA	33.7	‐
^[^ ^136^ ^]^	Ti_3_C_2_T*_x_* MXene	3.2	‐
	MoS_2_ on multilayer MXene (MoS_2_/MXI)	6.0	‐
	MoS_2_ on delaminated MXene (MoS_2_/MXII)	9.6	‐
^[^ ^102^ ^]^	Ti_3_C_2_T*_x_*	25.38	‐
	Cu_2_O	16.24	‐
	Ti_3_C_2_T*_x_* nanosheet (1%)/Cu_2_O	17.08	‐
	Ti_3_C_2_T*_x_* nanosheet (3%)/Cu_2_O	19.24	‐
	Ti_3_C_2_T*_x_* nanosheet (5%)/Cu_2_O	21.43	‐
	Ti_3_C_2_T*_x_* nanosheet (7%)/Cu_2_O	25.61	‐
	Ti_3_C_2_T*_x_* nanosheet (10%)/Cu_2_O	27.35	‐
^[^ ^143^ ^]^	Ti_3_C_2_T*_x_* MXene	13.54	‐
	Sodium alginate (SA)	9.23	‐
	Ti_3_C_2_T*_x_* MXene/sodium alginate (4:20)	9.66	‐
^[^ ^101^ ^]^	Ti_3_C_2_T*_x_* MXene	8.525	‐
	Ti_3_C_2_T*_x_* MXene/10% KH750 polymer	75.442	‐
^[^ ^85^ ^]^	Ti_3_C_2_T*_x_*	167.99	‐
	Chitosan based porous carbon microsphere (CPCM)	2433	‐
	CPCM/Ti_3_C_2_T*_x_* MXene	1878	‐

In another study, layered structures of Ti_3_C_2_T*_x_* MXene were prepared and modified with amino groups and then further treated with a borate affinity sorbent, 4‐formylphenylboronic acid (4‐FPBA), to produce Fe_3_O_4_@Ti_3_C_2_‐BA. BET analysis revealed that this nanocomposite possessed a high specific surface area of 33.77 m^2^ g^−1^ (Table 1).^[^ ^110^ ^]^

It has also been observed that heterogeneous nanoadsorbent composed of Ti_3_C_2_T*_x_* MXene nanosheets (MX) functionalized with nanolayered molybdenum disulfide (MoS_2_) exhibited relatively high adsorption kinetics the MoS_2_/MXII structure due to its high specific surface area of 9.6 m^2^ g^−1^ (Table 1). These nanostructures were formed by functionalizing MoS_2_ on stacked MXene layers and then subjecting them to ultrasonication to improve delamination. A relatively increased surface area through delamination onto a single or few layers of MXene provided access to metal ions to interact with surface moieties in the MoS_2_/MXII composite.^[^ ^136^ ^]^ Similar observations were made when Ti_3_C_2_T*_x_* MXene was functionalized with KH750 polymer, which resulted in increased surface area of the product from 8.525 to 75.442 m^2^ g^−1^ (for 10% KH750 polymer) (Table 1). The increase is assigned to the absorption and intercalation of KH750 into the layers of Ti_3_C_2_ which provided more interface and active site for adsorption and interfacial charge transfer capacity.^[^ ^101^ ^]^ Wu et al. prepared a pod‐inspired super‐adsorbent material (CPCM/MXene) from Ti_3_C_2_T*_x_* MXene and chitosan‐based porous carbon microspheres (CPCM). They observed a remarkable increase in the specific surface area of the MXene after functionalization with CPCM from 167.99 to 1878 m^2^ g^−1^ (Table 1). The observed tremendous increase was attributed to the presence of the CPCM, which provided abundant active sites for adsorption and interfacial interaction. The presence of mesopores and macropores enhanced a faster migration of pollutants to the interior of the adsorbent.^[^ ^85^ ^]^

#### 3.3.5 FTIR

The evidence of successful surface functionalization of MXene can be probed by FTIR. Pristine MXene shows characteristic peaks at 3435 cm^−1^ (—OH), 1641 cm^−1^ (C=O), 1406 cm^−1^ and 545 cm^−1^ (Ti—O). FTIR spectra of MXene/PEI/SA (MPA) composite aerogel, prepared by Feng et al., showed three new peaks at 669 cm^−1^ (N—H stretching vibrations), 1041 cm^−1^ (Si—O—Si), and 1118 cm^−1^ (Si—O—Si) (Figure 6d), confirming that the amino group and APTES were successfully fixed on the surface of MXene.^[^ ^117^ ^]^ Similarly, Kong et al. observed a change in the broadness of the —OH band, the emergence of a new —NH_2_ stretch, and weak bands at 2919 and 2851 cm^−1^ for CH_2_ stretches, for NH_2_—Ti_3_C_2_T*_x_*‐0.5. Complimentary peaks at 1535, 1040 and 939 cm^−1^ were also observed and attributed to —NH_2_ scissoring vibration, Si—O—Si and Ti—O—Si stretches, respectively (Figure 6e).^[^ ^118^ ^]^ In the case of Cui et al., where sodium alginate was used to functionalize Ti_3_C_2_T*_x_* MXene surface, FTIR spectra revealed peaks at 1034 and 821 cm^−1^ which were assigned to C—O and Na—O bonds, respectively (Figure 6f).^[^ ^143^ ^]^ The FTIR of L‐DOPA surface functionalized Ti_3_C_2_T*_x_* MXene, as shown in Figure 6g, revealed stretches at 2970 and 2900 cm^−1^ for to C—H antisymmetric stretching vibration for PDOPA, vibrations at 1048 cm^−1^ for C—O, and vibrations between 1200 and 1400 cm^−1^ for C—OH (of the catechol group in PDOPA).^[^ ^123^ ^]^

#### 3.3.6 TGA

TGA has been employed to provide evidence of the thermal stability of the functionalized samples.^[^ ^144^, ^145^ ^]^ Pristine MXene shows very low weight loss at 800 °C which indicates good thermal stability.

In a study, by Sun and his colleagues, where MXene‐PDA and MXene‐PDA‐IL were prepared by functionalizing Ti_3_C_2_T*_x_* MXene with polydopamine (PDA) and/or an ionic liquid via a combination of mussel inspired chemistry and Michael addition reaction, TGA data showed a weight loss percentage of 20.4% for MXene‐PDA and 41.2% for MXene‐PDA‐IL at 800 °C. The weight loss was attributed to the carbonization of organic matter. The paper also reported mass ratios of PDA and ionic liquid on MXene‐PDA and MXene‐PDA‐IL as 19.1% and 20.8%, respectively (Figure 6h).^[^ ^101^ ^]^ Lim and co‐workers employed TGA to estimate the amount of 2 different polyelectrolytes (PEs) grafted onto the surface of Ti_3_C_2_ MXene. The first, is an anionic random copolymer of 2‐acrylamido‐2‐methylpropane sulfonic acid (AMPS) and acrylic acid, poly(AMPS‐*co*‐AA). The second PE is a zwitterionic random copolymer of [2‐methacryloy(oxy)ethyl]dimethyl‐(3‐sulfopropyl)ammonium hydroxide (DMAPS) and the acrylic acid, poly(DAMPS‐*co*‐AA). The TGA data showed PE contents of ≈14.15 wt% for MXene‐g‐poly(AMPS‐*co*‐AA), and ≈21.58 wt% for MXene‐g‐poly(DMAPS‐*co*‐AA).^[^ ^146^ ^]^

The TGA data from the paper by Pandey et al., where MXene was functionalized/crosslinked with cellulose acetate (CA), showed that there was no appreciable weight loss in pristine cellulose acetate (CA), crosslinked CA(CCA), non‐crosslinked 10% MXene/CA (10% MXene/CA) and crosslinked 10% MXene @ CA (10% MXene@ CA). However, a three‐step weight loss was observed for all prepared samples. The first step at 150 °C was attributed to the release of adsorbed and bound water present on the membrane matrix. The second step between 250 and 375 °C was assigned to the decomposition of oxygen‐containing groups and the polymer backbone. The third step was assigned to the decomposition of residual carbon from CA and MXene, with CA polymer matrix decomposition starting from 266 °C. It was noted that upon the incorporation of 10% MXene, a significant improvement of thermal stability was observed when decomposition temperature further shifted to ≈300 °C. This phenomenon is linked to crosslinking of the CA polymer backbone with MXene (Figure 6i).^[^ ^112^ ^]^

Furthermore, TGA has been employed to identify bonding reaction temperature and to analyze the stages of weight loss for carbon nanotubes modified MXene (MXene‐CNT). Two weight‐loss steps were observed during this process; first occurring from 30 to 100 °C, associated with the evaporation of water within the interlayer spacing of MXene and CNTs, and second, from 100 to 200 °C, assigned to loss of combined water in the interlayer and water by bonding reaction between the surface groups of MXene nanosheets and CNTs. A small platform was also observed between 118 and 130 °C which was attributed to the bonding reaction as well (Figure 6j).^[^ ^115^ ^]^

#### 3.3.7 Raman Spectroscopy

Raman spectroscopy is an additional technique that provides information about the chemical structure, phase, crystallinity, and molecular interactions of the sample. Raman spectrogram from Wu's study on CPCM/MXene^[^ ^85^ ^]^ displayed modes at 1350 cm^−1^ (D band) and 1590 cm^−1^ (G band), respectively, referred to as the degree of defect disorder and graphitization of carbon materials (Figure 6k). After activation by thermal treatment at elevated temperature, the *I*_D_/*I*_G_ values of CPCM and CPCM@MXene became significantly higher than that of HCS indicating that they reached a higher degree of graphitization. In Jin's paper,^[^ ^111^ ^]^ Ti_3_C_2_T*_x_*/PmPD composite was prepared by modifying Ti_3_C_2_T*_x_* with poly(m‐phenylenediamine) (PmPD). Raman spectrogram of the Ti_3_C_2_T*_x_*/PmPD composite showed two strong peaks like that of PmPD at 1355 and 1558 cm^−1^, which indicates the strong interaction between PmPD and Ti_3_C_2_T*_x_* (Figure 6l).^[^ ^111^ ^]^

### 3.4 Techniques for Surface Modification and Functionalization of MXenes

To control and improve the chemical and structural stability of MXenes for specialized applications, tailored functionalization of the surface is required.^[^ ^124^, ^128^, ^148^, ^149^ ^]^ Meng et al. explored the effect of surface functionalities in adsorbing urea from an aqueous solution.^[^ ^124^ ^]^ In this work, the interaction between urea and MXene with three surface terminations (—OH, —O‐ or —F), was gleaned from first principles calculation. The most stable adsorption state of urea was via the —OH terminated surfaces, followed by —O‐ and F‐ terminated surfaces. The higher stability of urea on the —OH terminated surfaces was attributed to the difference in charge density.^[^ ^124^ ^]^ Functionalization of MXenes can be achieved using several techniques, that typically involved bottom‐up chemical treatment which includes hydrothermal,^[^ ^92^, ^136^, ^150^, ^151^ ^]^ solvothermal,^[^ ^133^, ^152^, ^153^ ^]^ CVD,^[^ ^145^, ^154^ ^]^ and plasma‐assisted synthetic routes.^[^ ^155^ ^]^

#### 3.4.1 Hydrothermal Method

**Figure 7:**
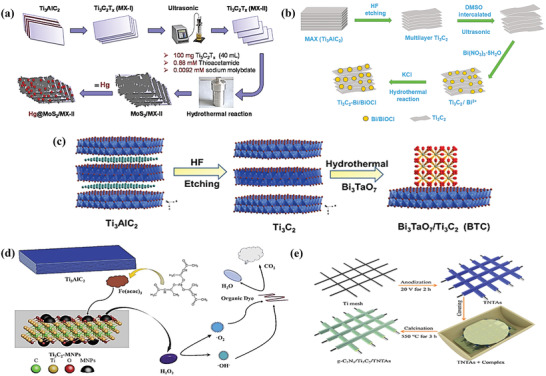
a) Diagram of procedure of hydrothermal synthesis employed in functionalizing MoS_2_ on Ti_3_C_2_ MXene sheets. Reproduced with permission.^[^ ^136^ ^]^ Copyright 2019, Elsevier. b) Schematic diagram of the solvothermal process employed in the preparation process of Ti_3_C_2_‐Bi/BiOCl composite. Reproduced with permission.^[^ ^172^ ^]^ Copyright 2020, Elsevier. c) Schematic formation of Bi_3_TaO_7_/Ti_3_C_2_ MXene sample by hydrothermal method. Reproduced with permission.^[^ ^113^ ^]^ Copyright 2020, Elsevier. d) Schematic diagram showing a novel one‐step strategy for the preparation of Fe_3_O_4_ nanoparticles on Ti_3_C_2_ MXene. Reproduced with permission.^[^ ^108^ ^]^ Copyright 2020, Elsevier. e) Schematic diagram of synthesis process involving the use of electrochemical anodization in producing g‐C_3_N_4_/Ti_3_C_2_ MXene/TNTAs. Reproduced with permission.^[^ ^172^ ^]^ Copyright 2020, Elsevier.

One of the many methods that have been explored for the synthesis of composite MXenes with surface functionalized properties is the hydrothermal method via a one‐pot synthesis approach.^[^ ^141^, ^142^, ^156^, ^157^, ^158^ ^]^ This technique has been used to introduce various crystalline nanoparticles onto the surfaces of MXenes. During the synthesis process, crystalline nanoparticles are rapidly formed from metal oxides undergoing hydrolysis and dehydration reactions as a function of temperature.^[^ ^159^ ^]^ Shahzad and co‐workers made use of the hydrothermal reaction in a Teflon‐lined autoclave to synthesize Ti_3_C_2_T*_x_* MXene (MX) nanosheets functionalized with nanolayered molybdenum sulfide at 180 °C for 12 h.^[^ ^136^ ^]^ During the synthesis, Ti_3_C_2_T*_x_* was sonicated using a probe sonicator for 30 min prior to reaction with the molybdenum precursor (**Figure** **7**a). Similarly, Li et al. reported the synthesis of Bi_3_TaO_7_/Ti_3_C_2_ where Ti_3_C_2_, Bi_3_TaO_7_ (prepared from Bi(NO_3_)_3_.5H_2_O) and TaCl_5_ were used as precursors. They transferred these precursors into a Teflon‐lined stainless‐steel autoclave and maintained a constant temperature of 180 °C to obtain the desired product (Figure 7c).^[^ ^113^ ^]^ SEM images reveal that the Bi_3_TaO_7_ nanoparticles covered the surface of the Ti_3_C_2_ nanosheets.

Cui et al. were the first to report the use of the hydrothermal method to incorporate Fe_3_O_4_ nanoparticles on Ti_3_C_2_ MXene nanosheets (Figure 7d). By using hydrazine hydrate as a reductant, ferric acetylacetonate as an iron source, and Ti_3_C_2_ nanosheets, dissociative Fe^3+^ was produced and reduced to Fe^2+^ on heating to 270 °C to give well dispersed Fe_3_O_4_ nanoparticles of ≈5 nm in size on Ti_3_C_2_ nanosheets.^[^ ^108^ ^]^ A simple in situ growth process has also been used to synthesize Fe_3_O_4_/MXene where Ti_3_C_2_ MXene nanosheets and precursor solution of Fe_3_O_4_ were dispersed in deionized water.^[^ ^160^ ^]^

#### 3.4.2 Solvothermal Method

Solvothermal processes are known for their simple operation, mild conditions, and capability to deliver copious quantities of product materials. Solvothermal synthesis is performed in sealed containers in which the solvents can be uniquely brought to a temperature above the boiling points by the increase of autogenous pressures.^[^ ^161^ ^]^ The products of solvothermal reactions are usually crystalline and do not require post‐annealing treatment.^[^ ^161^, ^162^ ^]^ This method has been successfully used to prepare 2D/2D heterojunction of Bi/BiOCl‐Ti_3_C_2_ (Figure 7b) which effectively improved the visible light absorption, charge separation, and transport.^[^ ^163^ ^]^

#### 3.4.3 Other Processing Techniques

Electrochemical anodization is a well‐established surface modification technique that is widely used because of its simplicity and reproducibility. Besides, the feasibility to tune the size and meet the demands of specific applications by means of controlled anodic oxidation of the metal substrates, makes this method attractive.^[^ ^164^ ^]^ During anodization, a constant voltage or current is applied between the anode and cathode, which stimulates Redox reactions and field‐driven ion diffusion to occur simultaneously.^[^ ^165^ ^]^ Although this technique has been available for a long time, it was not until the 1990s that researchers discovered that highly ordered nanoporous structures can be achieved by properly tuning anodization conditions including electrolyte composition and concentration, as well as temperature and anodization voltage.^[^ ^166^ ^]^

CVD has also been employed as a surface modification technique that involves the formation of a thin solid film on a substrate material by a chemical reaction of vapor‐phase precursors.^[^ ^167^, ^168^, ^169^ ^]^ The use of CVD on powders and other mediums of nanotechnology needs further processing such as individual particle treatment, efficient gas‐solid mixing, and confinement of submicron powders operating at sub‐atmospheric pressure.^[^ ^168^ ^]^ One of the best ways of treating powders by CVD is by maintaining them in a fluidized bed. Through this technique, a bed of solid particles over a gas distributing plate (called the grind) is made to behave like a liquid by passing gas through it at a flow rate above a certain critical value. Fluidized bed CVD (FBCVD) provides better finishing to other flat surfaces especially on wafers when compared to other deposition methods. It ensures satisfactory uniformity of deposition on each wafer, and from wafer to wafer, especially those running under low pressure.^[^ ^167^, ^170^, ^171^ ^]^ In a study conducted by Diao et al., ternary photocatalyst g‐C_3_N_4_/Ti_3_C_2_/TiO_2_ nanotube arrays (TNTAs) on Ti meshes was synthesized. The titanium nanotube arrays were prepared by electrochemical anodization (EA) followed by the preparation of the ternary structure; g‐C_3_N_4_/Ti_3_C_2_/TNTAs using CVD (Figure 7e). The final structure was annealed in a tube furnace at 550 °C for 3 h with a heating rate of 5 °C min^−1^.^[^ ^172^ ^]^

Given the surface tunability of MXenes, numerous studies involved integrating new surface groups which enhanced metal adsorption efficiency and selectivity.^[^ ^32^, ^173^ ^]^ The hydrophilicity and electrical properties of MXenes ensure an effective combination of photogeneration and charge separation phenomena as well as deionization for wastewater treatment.^[^ ^32^, ^174^, ^175^ ^]^

## 4 MXenes for Wastewater Treatment

**Figure 8:**
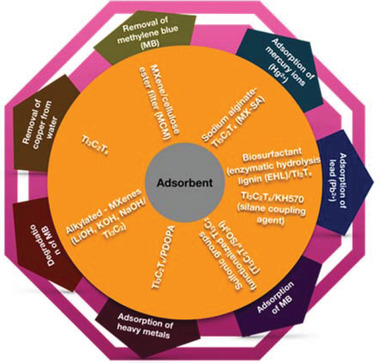
Various applications of surface functionalized MXenes used as adsorbent materials for wastewater treatment.

This section reviews the latest advances in the application of MXenes for wastewater treatment. So far, MXenes have been adopted mainly as adsorbents (**Figure** **8**) and as photocatalysts. Two emerging fields where MXenes find application are in antifouling/antibacterial systems and in the removal of radioactive waste. The advantages of MXenes surface functionalization will be discussed.

### 4.1 MXenes as Adsorbents

2D nanomaterials of which MXenes form a part have attracted increasing research attention over the last decade for adsorption applications.^[^ ^69^, ^70^ ^]^ Recent reports have demonstrated that MXenes and MXene based materials potential adsorbents for the removal of various environmental pollutants because of their environmentally benign characteristics.^[^ ^32^, ^33^, ^176^ ^]^ good structural and chemical stabilities,^[^ ^177^ ^]^ and hydrophilic surfaces.^[^ ^178^ ^]^ Titanium carbide (Ti_3_C_2_T*_x_*) has demonstrated the ideal characteristics as an adsorbent for the removal of some heavy metal ions including Pb(II), Cr(IV), Hg(II), Cd(II), and Cu(II).^[^ ^70^, ^179^, ^180^, ^181^, ^182^ ^]^ Adsorption is one of the effective methods of removing heavy metal ions where other methods such as biological and chemical processes cannot.^[^ ^183^, ^184^, ^185^, ^186^ ^]^ Ti_3_C_2_T*_x_* MXenes with the large specific surface area, hydrophilicity, and unique surface functional properties, allows it to adsorb metal ions through electrostatic and chemical interactions.^[^ ^187^ ^]^ The ability of the MXenes surface to hold an electrostatic charge is linked to the nature of the functional group present on its surface (‐F, ‐O, ‐OH), the presence of which is a contribution from the nature of the etchants and intercalants used in the etching, intercalation, and delamination process.^[^ ^180^, ^188^, ^189^ ^]^ These allow for electrostatic interactions with opposite charges of potential targets present in the wastewater. The MXenes surface can be negatively or positively tuned to adsorb charged targets by adjusting the pH of the solution (protonation/deprotonation of the surface).^[^ ^179^, ^190^, ^191^ ^]^ Targets of interest could include various heavy metals, dyes, radionuclides, or some contaminants that constitute targets in wastewater treatment.

**Figure 9:**
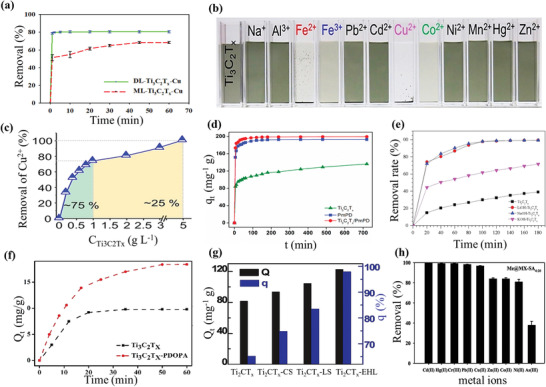
Surface modified MXene used as adsorbent a) delaminated Ti_3_C_2_T*_x_* MXene (DL‐ Ti_3_C_2_T*_x_*) showing better adsorptive properties as compared to multilayer Ti_3_C_2_T*_x_*. Reproduced with permission.^[^ ^179^ ^]^ Copyright 2017, American Chemical Society. b) Digital photograph on adsorption of different metal ions (1 mg L^−1^) on histidine His@TiO_2_@d‐ Ti_3_C_2_T*_x_* colloidal suspension (50 mg L^−1^) after 5min of contact and c) removal efficiency of Cu^2+^ ions versus different concentrations of His@TiO_2_ @ d‐ Ti_3_C_2_T*_x_* which shows ≈75% of Cu^2+^ ions was removed by the 1gL^−1^ His @TiO_2_@ Ti_3_C_2_T*_x_* MXene. Reproduced with permission.^[^ ^86^ ^]^ Copyright 2020, John Wiley and Sons. d) High adsorption efficiency of Cr(VI) with surface functionalized Ti_3_C_2_T*_x_* MXene with poly(m‐phenylenediamine). Reproduced with permission.^[^ ^206^ ^]^ Copyright 2019, Elsevier. e) Methylene blue removal rate of alkali treated MXenes (LiOH‐Ti_3_C_2_T*_x_* MXene, NaOH‐Ti_3_C_2_T*_x_*, and KOH‐ Ti_3_C_2_T*_x_*). Reproduced with permission.^[^ ^178^ ^]^ Copyright 2017, Elsevier. f) Comparison of Cu^2+^ adsorption between Ti_3_C_2_T*_x_* MXene and Ti_3_C_2_T*_x_* MXene surface functionalized with levodopa (amino acid). Reproduced with permission.^[^ ^123^ ^]^ Copyright 2019, Elsevier. g) Plot of adsorption performances of Ti_2_CT*_x_* MXene chitosan functionalized on Ti_2_CT*_x_* MXene (Ti_2_CT*_x_*‐CS), lignosulfonate functionalized on Ti_2_CT*_x_* (Ti_2_CT*_x_*‐LS), and enzymatic hydrolysis lignin functionalized on Ti_2_CT*_x_* MXene (Ti_2_CT*_x_*‐EHL). Reproduced with permission.^[^ ^205^ ^]^ Copyright 2019, Elsevier. h) Simultaneous adsorption for 8 toxic metal ions in a batch system (conditions: 50 mg adsorbent dose added to 30 mL aqueous solution containing 3 ppm of each metal ion agitated. Reproduced with permission.^[^ ^147^ ^]^ Copyright 2019, Elsevier.

Copper (Cu) has been identified as an essential element involved in several physiological processes, however, problems arise when Cu is found in excess in food or water. This leads to health problems like cardiovascular disease, liver failure, kidney dysfunction, gastrointestinal illnesses, nausea, copper homeostasis, liver toxicity to both human and aquatic life with short‐ and long‐term acute exposure, oxidative stress‐related mineral contents (mine waste), the pH of the water and common plumbing system (due to oxidation of copper pipelines).^[^ ^179^ ^]^ Adsorption has been adapted as a preferred solution to remove copper ions from water because the process involved is simple cost‐effective.^[^ ^192^, ^193^, ^194^, ^195^ ^]^ In 2017, Shahzad and co‐workers produced delaminated (DL)‐Ti_3_C_2_T*_x_* MXene material with strong Cu ion adsorption and a large uptake capacity.^[^ ^187^ ^]^ The nanosheets created were pH‐dependent, demonstrating low adsorption at lower pH values (< 2.5) – reflecting a change in the surface charge of the adsorbent due to competition between the H^+^ and Cu^2+^ ions for access to the surface sites. The presence of excess H^+^ ion under acidic conditions results in the protonation of the hydroxyl terminal groups on the Ti_3_C_2_T*_x_* surface, making them positively charged, thus reducing Cu^2+^ ions uptake. However, the maximum removal efficiency achieved for Cu^2+^ ions was 80% (corresponding to 78.45 mg g^−1^ adsorption capacity) in 60 s at an optimal pH of 5 (**Figure** **9**a).^[^ ^179^ ^]^ In another study, the adsorption of Cu^2+^ ions from an aqueous solution was realized using a rutile phase of titanium dioxide formed on delaminated Ti_3_C_2_T*_x_* MXene and functionalized with histidine (His@TiO_2_ @d‐ Ti_3_C_2_T*_x_*).^[^ ^86^ ^]^ The estimated adsorption capacity for Cu^2+^ was 95 mg g^−1^ corresponding to a removal efficiency of 75% in 5 min (Figure 9c). However, the as‐synthesized His@TiO_2_ @d‐ Ti_3_C_2_T*_x_* materials showed a reduction in Cu^2+^ ion adsorption as the initial Cu^2+^ concentration increased. This was attributed to the fact that restacking of MXene flakes reduce the number of available active sites as well as the surface area to volume ratio. His@TiO_2_ @d‐ Ti_3_C_2_T*_x_* also showed good degrees of adsorption toward Fe^2+^, Fe^3+^, Cu^2+^, and Co^2+^ as shown in Figure 9b.^[^ ^86^ ^]^

Another significant and highly toxic, carcinogenic, and mutagenic heavy metal water pollutant is Cr(VI), and its adsorptive behavior on MXenes has been investigated.^[^ ^111^ ^]^ In a more recent study by Feng et al., MXene/PEI modified sodium alginate aerogel showed good adsorption capacity of 538.97 mg g^−1^ for Cr(VI) removal, an ultrahigh adsorption capacity of 3568 mg g^−1^ toward Congo Red. The unique adsorption properties of the composite are ascribed to strong electrostatic attraction and the synergetic effect of surface adsorption and intercalation adsorption. Furthermore, the material showed outstanding antibacterial properties against *S. aureus* and *E. coli*. The adsorption data fitted well with the Langmuir adsorption isotherm and the pseudo‐second‐order kinetic model.^[^ ^117^ ^]^ In another work by G. Yang et al., a novel imidazoles‐MXene hybrid composite (Ti_3_C_2_@IMIZ), showed high adsorption affinity for Cr(VI). The adsorption behavior and process analysis show that the adsorption mechanism is mainly physical adsorption through electrostatic interaction.^[^ ^106^ ^]^

Amino‐functionalized MXenes (NH_2_‐Ti_3_C_2_T*_x_*) were observed to have strong selective adsorption and reduction ability for Cr(VI) ions in an aqueous solution. The use of density functional theory calculations revealed that the synergy between Ti and N remarkably boosts the binding energy of MXene toward Cr(VI) along with the electron density on the MXene surface. The maximum adsorption capacities for Cr(VI) onto optimized NH_2_‐ Ti_3_C_2_T*_x_* calculated from the Langmuir model was 107.4 mg g^−1^.^[^ ^118^ ^]^ Linfeng Jin et al. also reported that MXene functionalized with poly(m‐phenylenediamine) (Ti_3_C_2_T*_x_*/PmPD) contributed to the enhanced adsorption of Cr(VI). The maximum Cr(VI) adsorption by Ti_3_C_2_T*_x_*/PmPD was 540.47 mg g^−1^ which was superior to both pure PmPD (384.73 mg g^−1^) and pure Ti_3_C_2_T*_x_* (137.45 mg g^−1^), shown in Figure 9d. The enhanced performance is attributed to the synergistic effects between Ti_3_C_2_T*_x_* MXene and PmPD. The Cr(VI) removal efficiency still remained 90% after five rounds.^[^ ^111^ ^]^

Alkalized MXenes have also been shown to possess significantly improved adsorption capacity for toxic metal ions. Ti_3_C_2_T*_x_* is activated or alkalized by treatment in a hot alkaline solution of LiOH, NaOH, and KOH.^[^ ^178^ ^]^ This treatment causes an increase in interlayer spacing, which results in higher catalytic activity and enhanced adsorption performance of the Alkalized MXenes. EDS analysis has been employed to confirm the successful reduction in the number of F atoms on the surface of MXenes and the subsequent increase in O atoms reflecting the increase in —OH groups, which readily adsorb small molecules or ions in solution.^[^ ^178^, ^196^ ^]^ Zheng et al. speculated that more —OH groups rendered better adsorption for alkali‐Ti_3_C_2_T*_x_* as demonstrated from their preparation of 3 types of aqueous solution (LiOH, NaOH, and KOH). Each exhibited faster methylene blue (MB) removal rates when compared with pristine Ti_3_C_2_T*_x_* since the terminal —OH groups readily adsorb cationic dyes.^[^ ^178^ ^]^ Amongst these three MXene adsorbents, NaOH‐Ti_3_C_2_T*_x_* had the highest adsorption capacity of 189 mg g^−1^ for MB (Figure 9e) followed by LiOH‐Ti_3_C_2_T*_x_* (121 mg g^−1^) and KOH‐Ti_3_C_2_T*_x_* (79 mg g^−1^).^[^ ^178^ ^]^ The performance of NaOH‐Ti_3_C_2_T*_x_* is comparable to other 2D materials such as graphene oxide^[^ ^197^ ^]^ and MoS_2_.^[^ ^182^ ^]^

Another way to improve the adsorption of toxic metals is by increasing the number of active sites as demonstrated by Zhang et al. by creating 2D Ti_2_C MXene nanosheets on a commercialized mixed cellulose ester filter paper (MCM) with a pore size of 0.22 µm.^[^ ^216^ ^]^ The materials showed complete dye removal of methylene blue solution (90 mg L^−1^). They reported that the rejection of dyes by these materials started after adsorptive sites on the surface were occupied, and this phenomenon was attributed to the presence of small d‐spacing (1.41 nm) of MXene nanosheets in the MCM.^[^ ^198^ ^]^ Gan and co‐workers adopted a simple one‐step inspired route to modify the surface of Ti_3_C_2_T*_x_* MXenes to form polymeric composites by mixing MXenes (Ti_3_C_2_T*_x_*) and 3,4‐dihydroxyphenylalanine (l‐DOPA) under mild reaction conditions such as room temperature, benign solvent, alkaline environment, and absence of catalysts. The result was a Ti_3_C_2_T*_x_*_‐_PDOPA composite with abundant carboxyl groups on the surface of the Ti_3_C_2_T*_x_* MXenes and was used to investigate Cu^2+^ adsorption.^[^ ^123^ ^]^ They observed that for Cu^2+^ concentration increasing from 5 to 30 mg L^−1^, the adsorption capacity of Ti_3_C_2_T*_x_*‐PDOPA increased from 10.5 to 33.5 mg g^−1^, confirming a concentration dependency. A Langmuir isotherm model added further support to these results, indicating a higher adsorption capacity of 65.126 mg g^−1^, thus possessing immense potential for wastewater treatment. Furthermore, the influence of pH on Cu^2+^ removal efficiency was investigated. The data showed that the adsorption capacity of Ti_3_C_2_T*_x_*‐PDOPA for Cu^2+^ at equilibrium increases from 2.6 to 46.6 mg g^−1^ when the pH of the solution increased from 1 to 11 and decreases to 38.8 mg g^−1^ at higher pH values of 11 to 13 (Figure 9f). It is expected that at low pH there would be competitive adsorption between H^+^ and Cu^2+^ and given the nature of the functional groups on the surface of Ti_3_C_2_T*_x_*‐PDOPA, both carboxyl and amine groups would be highly protonated, resulting in electrostatic repulsion of Cu^2+^, and a lower Cu^2+^ adsorption capacity from aqueous solution.^[^ ^123^ ^]^

Apart from Cu^2+^ ions, Pb^2+^ ions are also a significant source of pollution in water and lead to high toxicity and bioaccumulation.^[^ ^199^, ^200^ ^]^ These heavy metal ions are released into water bodies as a contribution from mining industries, battery manufacture, and historically, gasoline.^[^ ^194^ ^]^ Various adsorbents have been proven to be effective in removing heavy metal ions^[^ ^201^ ^]^ and among these, surface functionalized Ti_3_C_2_T*_x_* MXenes have been reported to show improved adsorptive properties.

Surface functionalization of Ti_2_CT*_x_* with biosurfactant has been shown to improve adsorption for Pb^2+^ ions. Wang et al. functionalized Ti_2_CT*_x_* with three different biosurfactants (chitosan, lignosulfonate, and enzymatic hydrolysis lignin) and demonstrated that the enzymatic hydrolysis lignin (EHL) functionalized Ti_2_CT*_x_* displayed the highest performance with a maximum Pb^2+^ ions adsorption capacity of 232.9 mg g^−1^. It was suggested that the use of non‐ionic EHL increased the number of active sites and the ion exchange efficiency of the resulting functionalized materials, resulting in the observed enhanced adsorption performance.^[^ ^202^ ^]^ Another way to modify Ti_3_C_2_T*_x_* powder and enhance its ability to adsorb organic pollutants and heavy metals include functionalizing these powders with the silane coupling agent KH570.^[^ ^101^ ^]^ This has been proven to be an effective way for the adsorption of Pb^2+^ ions. The adsorption capacity of Ti_3_C_2_T*_x_*‐KH570 powder for Pb^2+^ ion was determined to be 147.29 mg g^−1^, three times higher than that of pristine Ti_3_C_2_T*_x_* (48.28 mg g^−1^), at a temperature of 30 °C. The enhanced Pb^2+^ adsorption was attributed to the presence of MXene hydroxyl groups and KH570 carbonyl group of the methacryloxypropyl chain. One interesting feature about these materials is that they maintained an adsorption capacity of ≈110 mg g^−1^ after four cycles.^[^ ^101^ ^]^

MXene core (Ti_3_C_2_T*_x_*) shell aerogel spheres (MX‐SA) have been fabricated using Ti_3_C_2_T*_x_* MXene (MX) and sodium alginate (SA) for Hg^2+^ removal.^[^ ^147^ ^]^ These were prepared at different weight ratios (%w/w) by deposition into calcium chloride aqueous solutions (with Ca^2+^ ion serving as a crosslinking agent). The material exhibited 100% adsorption efficiency with a capacity of 932.84 mg g^−1^ for Hg^2+^, the highest adsorption capacity reported so far for adsorbents compared to 2D graphene oxide and its derivatives (with 34.63% efficiency) under highly acidic conditions. The adsorbent exhibited high single and multi‐component removal efficiencies with 100% efficiency for Hg^2+^ and > 90% efficiency for five other heavy metal ions namely Cd(II), Hg(II), Cr(III), Pb(II), Cu(II) (Figure 9g,h).^[^ ^205^ ^]^ The inner surface complexation between [Ti—O]‐ H^+^ and Hg^2+^ and ion exchange reaction between Ca^2+^ and Hg^2+^ are believed to be involved in the adsorption of Hg^2+^. The binding groups [Ti—O]—H^+^ and [Ti—O]—Ca^2+^ showed a metal‐ligand interaction with Hg^2+^.^[^ ^147^ ^]^ In another study on Hg^2+^ adsorption, a 2D Ti_3_C_2_T*_x_* MXene nanosheets (MX) functionalized with nanolayered molybdenum disulfide was successfully applied in the selective removal of toxic Hg^2+^ ions in water and elemental mercury in vapor form.^[^ ^136^ ^]^ It was observed that ultrasonication increased the surface area and interlayer distance of the Ti_3_C_2_T*_x_* nanosheets, which resulted in the enhanced removal capacity of mercuric ions by the composite. In addition, the adsorption data fitted well with the Langmuir adsorption isotherm and revealed a maximum adsorption capacity of 7.16 mmol g^−1^.^[^ ^136^ ^]^

Sulfonic acid‐functionalized Ti_3_C_2_ MXenes synthesized through a one‐step method using a sulfonated arenediazonium salt to introduce the sulfanilic moiety onto Ti_3_C_2_ sheets was used for methylene blue (MB) removal.^[^ ^127^ ^]^ The adsorption capacity of Ti_3_C_2_—SO_3_H for MB reached 111.11 mg g^−1^ compared to pristine Ti_3_C_2_ (21.10 mg g^−1^). This could be attributed to the electrostatic interaction between negatively charged adsorbent and cationic MB. It was also noted that dye adsorption capacity is more favorable when the aqueous solution is alkaline and more SO^3−^ groups are available on the surface of the adsorbent thus enhancing the electrostatic attraction between negatively charged adsorbent and the cationic dye. This can facilitate the combination of sorbent and adsorbate, thus improving the amount of dye uptake and adsorption. To add to this, it was concluded that the process of MB adsorption onto the surface of adsorbents was endothermic and spontaneous.^[^ ^127^ ^]^ Interestingly the authors did not address the role of Van der Waals interactions that may also be at play between the sulfanilic and MB arene moieties, particularly at low pH. An in situ method was employed to grow Fe_3_O_4_ particles onto the surface of phenylboronic acid modified Ti_3_C_2_T*_x_* nanosheets produced a novel magnetic borate modified MXene composite with high selective recognition and high adsorption properties for catecholamines and dopamine, respectively. The excellent adsorption property is assigned to the unique 2D layered structures, which helps to shorten the diffusion path and facilitate molecular transport. A high adsorption capacity of up to 319.6 mmol g^−1^ for dopamine and the adsorption of catecholamines was accomplished within 2.0 min.^[^ ^110^ ^]^ Functionalized cellulose/MXene composite aerogel (P‐M/MX‐m) with excellent adsorption performance for MB was prepared by the oxidative self‐polymerization of dopamine hydrochloride and freeze‐drying. Using the Langmuir isotherm model, the maximum adsorption capacity for MB reached 168.93 mg g^−1^. It was also established that a high concentration of chloride (>3%) enhanced MB removal.^[^ ^189^ ^]^

Various nanomaterials have been considered in applications such as water remediation. Some articles considered the use of in situ remediations where a pollutant scavenger is directly injected into the contaminant subsurface site. This injection enables active treatment of contamination sources and prevents recontamination at a low cost. Sehyeong Lim and co‐workers were the first to report on engineered Ti_3_C_2_ MXenes that had the ability to scavenge aquatic pollutants directly from the subsurface environments. The key idea is to chemically graft highly salt‐resistant polyelectrolytes (PEs) which can remain hydrated even in extreme saline environments. The PE grafted MXene retained a satisfactory adsorption capacity of ≈68 mg g^−1^ for MB as a model aqueous organic pollutant which was comparable to those of conventional adsorbents.^[^ ^136^ ^]^ In addition, novel heterogeneous structure of the GO/MXene composite membranes with properties that overcome the present limitations of low flux and instability associated with GO membranes. Optimized GO/MXene membrane possessed a permeation flux of 71.9 L m^−2^ h^−1^ bar^−1^ with a thickness of 550 nm, which was ten times the flux of pure GO membrane (6.5 L m^−2^ h^−1^ bar^−1^). The excellent water flux of the GO/MXene composite membrane compared to that of GO membrane was ascribed to the moderate increase in interlayer spacing of the membrane and the decrease of oxygen‐containing functional groups.^[^ ^191^ ^]^

A multidimensional MXene‐carbon nanotube (CNT) ultrathin membranes were prepared by loading a MXene intercalated with CNTs onto a tubular ceramic membrane.^[^ ^116^ ^]^ Taking advantage of the modes of Van der Waals interactions and repulsion between the MXene and functionalized CNTs, the 1D CNTs are well dispersed and intercalated into the 2D MXene nanosheets, resulting in a uniform network and continuous 3D labyrinthine short mass transfer channels, which can considerably improve the permeability and rejection performance of the membranes. Furthermore, 50‐h long‐term operation studies indicate the potential anti‐swelling property and stability of the MXene‐CNT membranes.^[^ ^116^ ^]^

Dopamine‐functionalized graphene oxide (DGO) nanosheets were intercalated into Ti_3_C_2_T*_x_* nanosheets via vacuum filtration on hydrophilic polyvinylidene fluoride (PVDF) membranes (used as support layer).^[^ ^203^ ^]^ The addition of DGO was observed to increase the mechanical stability of the composite membrane but reduced the interlayer spacing. The optimized composite membrane (MXene: DGO = 1:2) was ≈2 µm thick and exhibited an excellent dye rejection ratio of 98.1% (for Direct Red 28) and 96.1% (for Direct Black 38) and possessed a high‐water flux value of 63.5 Lm^−2^ h^−1^ at a pressure of 0.1 MPa, compared with the pure MXene and DGO membranes. Furthermore, the optimized nanocomposite showed a relatively low rejection ratio for Na^+^ (9.7%) and Mg^2+^ (4.3%).^[^ ^203^ ^]^

A novel dopamine functionalized MXene (PDA@MXene/CA) with ultra‐high permeability and good selectivity was successfully synthesized and was found to have good hydrophilicity, and pure water flux of 271.2 Lm^−2^ h^−1^, which was 277% more than the unmodified membrane. An improvement in dye separation capability was observed – the rejection ratio of direct red 28 was 88.9%, and that of direct black 38 was 88.6%. PDA@MXene/CA was also noted to possess strong anti‐fouling characteristics and good antibacterial ability.^[^ ^204^ ^]^

Overall, MXenes show outstanding adsorptive properties for metal ions and non‐ionic atoms which are stronger than other 2D materials like graphene.^[^ ^31^ ^]^ These unique adsorption properties are generally attributed to the natural surface‐functionalization of MXenes during the synthetic process using acidic fluoride‐containing solutions.^[^ ^205^ ^]^

### 4.2 MXenes as Photocatalysts

**Figure 10:**
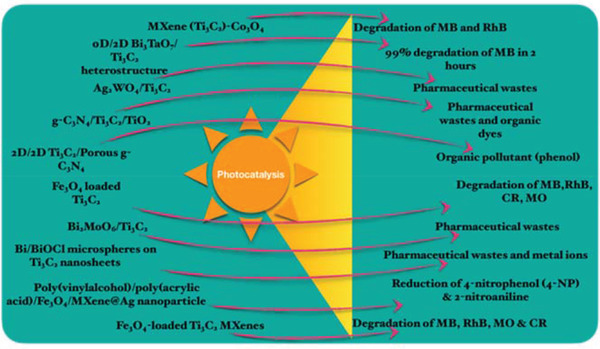
Various applications of MXenes used as photocatalyst materials for wastewater treatment.^[^ ^102^, ^108^, ^113^, ^163^ ^]^

Photocatalysis has over the years received great attention as an advanced catalysis technology with enormous potential for solving environmental and energy‐related challenges. The mechanism of photocatalysis involves i) the generation of charge carriers (electrons and holes) by photocatalyst after absorbing incident light energy; (ii) separation and migration of the photogenerated charge carrier to the surface of the photocatalyst; and (iii) the reduction and oxidation reactions by consuming photogenerated electrons and holes, respectively.^[^ ^187^, ^207^, ^208^, ^209^ ^]^ Among these three significant steps, the effective separation and migration of photogenerated charge carriers is the rate‐determining step. This step helps to prevent the recombination of photogenerated electrons and holes because recombination severely hinders photocatalytic activity and solar conversion efficiency.^[^ ^209^, ^210^, ^211^ ^]^ Scientists have, therefore, focused attention on fabricating photocatalysts that can efficiently suppress the recombination of charge carriers. To this end, surface factionalized MXenes are perceived as a solution to this bottleneck because of their excellent electrical properties that enhance charge separation efficiency.^[^ ^198^ ^]^ MXenes are also a promising co‐catalyst for excellent photocatalysts that work effectively under sunlight (**Figure** **10**) due to; i) the abundant functional groups from wet chemical etching process bonds MXene and other semiconductors well ii) the bandgap alignment of MXene can be modulated by tuning the surface chemistry iii) the conductive metal cores in the layered structure endows MXene with excellent metallic conductivity and electron acceptance.^[^ ^212^, ^213^, ^214^, ^215^, ^216^, ^217^, ^218^ ^]^ The synthesis methods mentioned earlier in this paper have been used to tune the surface chemistry of pristine MXene to enhance the separation efficiency of photogenerated charge carriers, acting as robust support, and enhancing adsorption.^[^ ^213^ ^]^

**Figure 11:**
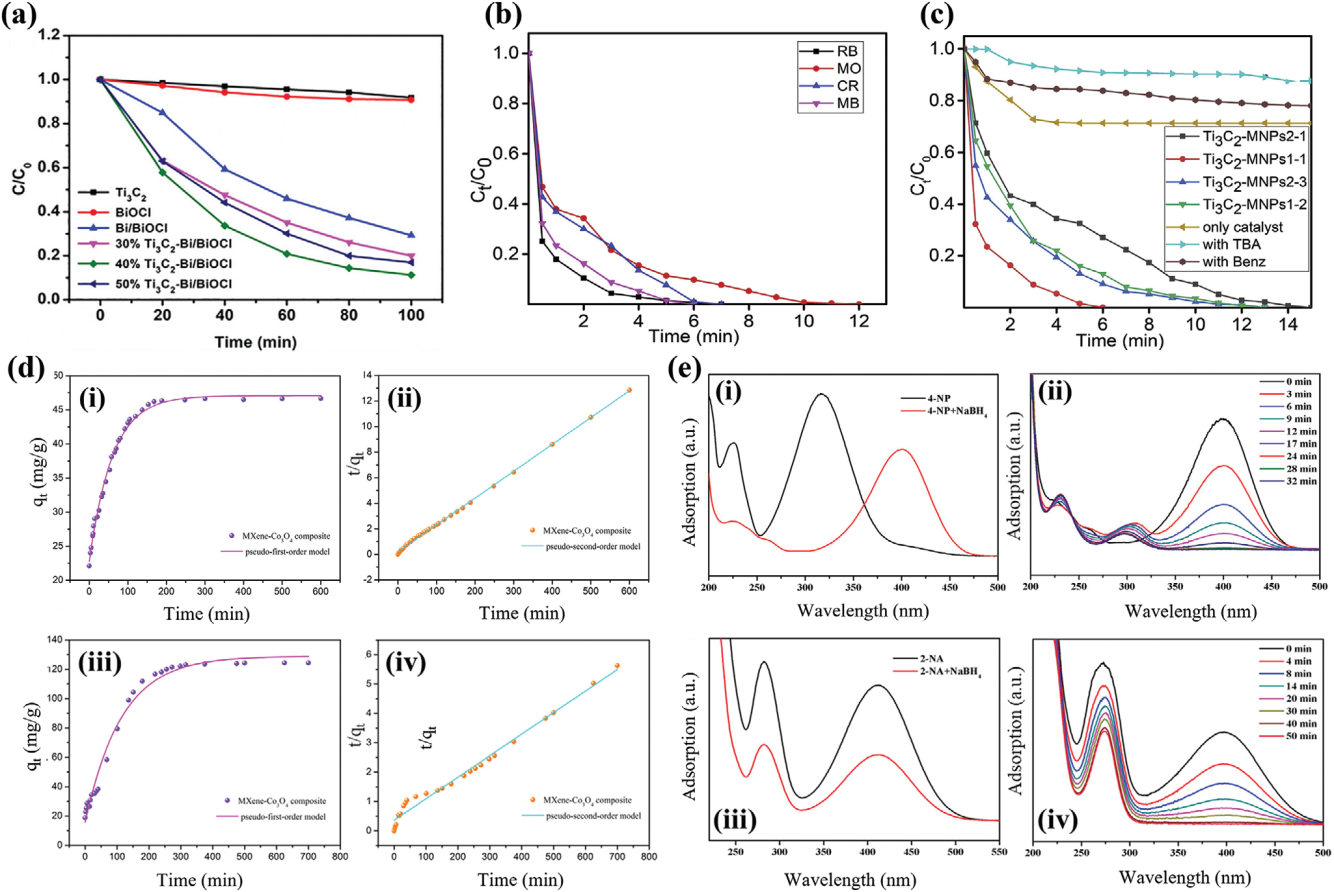
a) Photocatalytic degradation of ciprofloxacin over surface functionalized Ti_3_C_2 _MXene with Bi/BiOCl. Reproduced with permission.^[^ ^163^ ^]^ Copyright 2020, Elsevier. b) Photocatalytic degradation efficiencies of Fe_3_0_4_‐Ti_3_C_2_ MXene on four organic dyes (rhodamine B, methyl orange, congo red, methylene blue) and c) degradation efficiencies of Ti_3_C_2_‐MNPs with different mass ratios and degradation efficiencies with only Ti_3_C_2_‐MNPs. Reproduced with permission.^[^ ^108^ ^]^ Copyright 2020, Elsevier. d) adsorption kinetic curves of as‐prepared Ti_3_C_2_ MXene—Co_3_O_4_ nanocomposite on i,ii) rhodamine B and iii,iv) methylene blue. Reproduced with permission.^[^ ^152^ ^]^ Copyright 2019, ACS. e) i,iii) UV–vis spectra of 4‐nitrophenol (4‐NP) and 2‐nitroamiline (2‐NA) before and after adding NaBH_4_ aqueous solution, ii, iv) UV– vis spectra for the photocatalytic reduction of 4‐NP and 2‐NA with polyvinyl/polyacrylic/Fe_3_O_4_/Ti_3_C_2_T*_x_* MXene@AgNP20 composite nanofibers. Reproduced with permission.^[^ ^224^ ^]^ Copyright 2019, ACS.

One of the recent works reports the use of the precipitation method to prepare Ti_3_C_2_T*_x_*‐ nanosheets/Cu_2_O composite photocatalysts with a Schottky heterojunction was prepared for the decomposition of tetracycline (TC) antibiotics under visible light.^[^ ^102^ ^]^ The optimized Ti_3_C_2_T*_x_*‐nanosheets/Cu_2_O composite degraded 97.6% of TC in 50 min. The enhanced efficiency was attributed to superoxide radical (O^2•^) and hole (h^+^). Furthermore, the presence of a Schottky heterojunction contributed to the effective separation of electron‐hole pairs.^[^ ^102^ ^]^ Ti_3_C_2_ nanosheets were also introduced into Bi/BiOCl to produce a Ti_3_C_2_‐Bi/BiOCl composite. The photoactivity of the as‐synthesized materials was used to degrade the antibiotic, ciprofloxacin (CIP) present in water. Results demonstrated enhanced CIP degradation efficiency (89%) in comparison to BiOCl (10%) and Bi/BiOCl (≈71%) as shown in **Figure** **11**a.^[^ ^163^ ^]^ The increase in photocatalytic activity was attributed to the ability of Ti_3_C_2_ to form heterostructures which promotes the separation efficiency of photogenerated charge carriers.^[^ ^163^ ^]^ These results were verified from the ultraviolet‐visible diffuse reflectance spectrogram (UV–vis DRS) which showed that pristine BiOCl exhibited an absorption edge at ≈375 nm corresponding to 3.8 eV from the Tauc's plot, signifying higher bandgap and lower absorption of visible light. Remarkably, depositing Bi‐metal showed a stronger visible light absorption and is attributed to a surface plasmon resonance (SPR) effect. On the other hand, Ti_3_C_2_‐Bi/BiOCl composite demonstrated an obvious red shift into the visible region. Consequently, the as‐prepared material not only exhibited enhanced CIP degradation efficiency but also a significantly improved CIP degradation rate which was ≈22.3 and 1.76‐fold greater than that of BiOCl and Bi/BiOCl, respectively.^[^ ^163^ ^]^

Fe_3_O_4_ nanoparticles incorporated into Ti_3_C_2_ MXene nanosheets (Ti_3_C_2_‐MNPs) via an alkaline hydrothermal treatment showed an impressive activity toward the degradation of MB, rhodamine B (RhB), methyl orange (MO), and Congo red (CR) (Figure 11b,c).^[^ ^108^ ^]^ The degradation efficiency of these Ti_3_C_2_‐MNPs was investigated under specific experimental conditions: a dye concentration of 0.5 gL^−1^, in the presence of H_2_O_2_ (50 mm) at pH 3, a temperature of 40 °C, and a reaction time of 30 s. Under these conditions, the degradation efficiencies of RB, MO, CR, MB were 74.82%, 53.07%, 57.29%, and 67.77%, with completion times of 7, 12, 7, and 6 min, respectively.^[^ ^108^ ^]^

An in situ solvothermal method was used to synthesize Ti_3_C_2_ MXene‐Co_3_O_4_ nanocomposites that displayed excellent adsorption and catalytic degradation of rhodamine B molecules (Figure 11d‐i,ii) and MB (Figure 11d‐iii,iv).^[^ ^152^ ^]^ The removal rates of MB and RhB were stabilized in ≈240 and 80 min, respectively, indicating that the prepared complex acted as an effective dye adsorbent. These features could be attributed to the larger specific surface area which is very advantageous for the adsorption of dyes for catalytic requirements. In addition to the exceptional absorption and photodegradation properties, these nanocomposites also exhibited good stability and repeatability, reflected by good catalytic properties after 8 consecutive cycles of MB and RhB degradation.^[^ ^152^ ^]^ Fang and his colleagues for the first time, successfully prepared Ag_2_WO_4_/Ti_3_C_2_ Schottky junction photocatalyst by an electrostatically driven in situ growth strategy. The Ag_2_WO_4_/Ti_3_C_2_ showed good photocatalytic removal rates for tetracycline hydrochloride (62.9%) and sulfadimidine (88.6%). The photocatalytic performance was attributed to the formation of the Schottky heterojunction.^[^ ^219^ ^]^

Nanocomposite fibers prepared from mixing poly(vinyl alcohol), poly(acrylic acid), Fe_3_O_4_, and MXene via electrospinning technology and subsequently modified with Ag nanoparticles [PVA/PAA/Fe_3_O_4_/MXene @ AgNP] exhibited excellent photocatalytic properties under the ultraviolet spectrum at 25 °C for reduction of nitro compounds; 2‐nitroaniline (2‐NA) and 4‐nitrophenol (4‐NP). Both nitro compounds are present in water bodies due to anthropogenic activities and are harmful to humans and the environment because of their high solubility and toxicity. Catalytic activity was studied by placing the PVA/PAA/Fe_3_O_4_/MXene @ AgNP in an aqueous solution of NaBH_4_ with 2‐NA/4‐NP and the reducibility was measured using the UV–vis spectroscopy at room temperature. Figure 11(i,iii) represents the UV–vis spectra of 4‐nitrophenol (4‐NP) and 2‐nitroamiline (2‐NA) before and after adding NaBH_4_ aqueous solution. It is evident that 2‐NA and 4‐NP showed no color change after 24 h without a catalyst, however, on adding the nanocomposite fibers, the catalytic reaction for 4‐NP and 2‐NA was completed within 90 min (Figure 11e‐iii,iv).^[^ ^220^ ^]^ The study showed that the combination of MXene with PAA and PVA aided in the dispersion and surface area of MXene sheets, enhancing the active sites for the reduction and loading of AgNPs. In a recent work published by Li et al., a 0D/2D Bi_2_TaO_7_/Ti_3_C_2_ heterojunction, synthesized using a hydrothermal method exhibited 99% degradation of MB after 120 min under visible light as compared to pristine Bi_3_TaO_7_ with 40% degradation efficiency. ≈85% colorless phenol was also degraded and the material was also found to possess excellent stability up to 10 cycles under visible light irradiation.^[^ ^113^ ^]^

A faster photodegradation was observed for a 2D bismuth molybdate that was in situ grown on the surface of Ti_3_C_2_ MXene nanosheets to form Bi_2_MoO_6_/Ti_3_C_2_ MXene (BT‐X, where x is the weight percentage) via a one‐step hydrothermal route.^[^ ^221^ ^]^ The optimized photocatalyst BT‐30 exhibited the highest removal efficiency of tetracycline, TC (99%) within 30 min whiles the degradation rate of bare Bi_2_MoO_6_ was only 37%. It was observed that the reaction rate of BT‐30 decreased indicating that the photodegradation of TC increased with increasing Ti_3_C_2_ MXene content. BT‐30 showed the fastest reaction rate, ≈8.8 times faster than that of pristine Bi_2_MoO_6_, in degrading TC (*k* = 0.143 min^−1^). The optimized catalyst was also tested for Cr(VI) reduction and showed 99% reduction after 60 min.^[^ ^221^ ^]^

### 4.3 MXenes as Antibiofoulants/Antibacterial Agents

According to World Health Organization (WHO), water contamination and antimicrobial resistance (AMR) for pathogenic microbes are leading global development and health threats facing society.^[^ ^222^ ^]^ It is one of the topics of growing public health concern throughout the world as this greatly affects sustainable societal development and activities. However, it can be controlled through good water management. A number of novel methods (e.g., ultrafiltration, electrodialysis, reverse osmosis, forward, osmosis, membrane bioreactors, nanofiltration, microfiltration, and membrane distillation) have already been developed for water purification.^[^ ^223^ ^]^ One of the emerging and promising techniques for water purification applications is solar‐driven interfacial evaporation^[^ ^224^, ^225^, ^226^ ^]^ which localizes heat from solar radiation at the liquid surface by using photothermal materials (i.e., which convert solar light to heat efficiently^[^ ^209^ ^]^ (e.g., Mxene based materials,^[^ ^119^, ^227^, ^228^ ^]^ polymers,^[^ ^229^ ^]^ plasmonic metals,^[^ ^230^, ^231^, ^232^ ^]^ carbon‐based light‐absorbing materials,^[^ ^233^, ^234^, ^235^ ^]^ etc.) at the water‐air interface and generate steam. This method exhibiting, portability, reduced energy consumption, and higher efficiency is a green and sustainable technique for water purification.^[^ ^236^ ^]^

**Figure 12:**
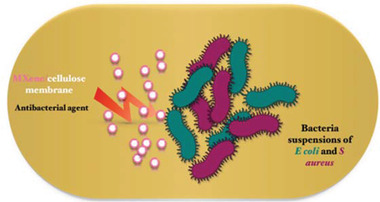
Novel Ti_3_C_2_ MXene on cellulose membrane.

**Figure 13:**
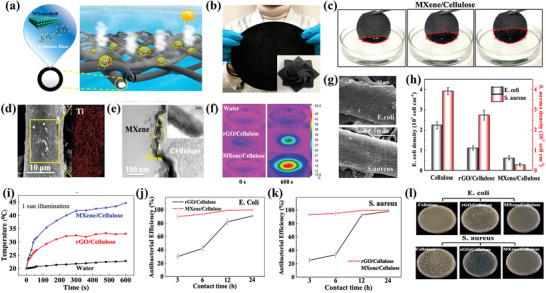
a) Schematic demonstration of the concept design for the MXene/cellulose anti‐biofouling membrane steam generator; b) Picture of MXene/cellulose membrane having 15 cm diameter and 0.2 mm thickness (inset shows the image of the flower based on the MXene/cellulose membrane); c) Water treatment shows the wetted part from bottom area to top part of MXene/cellulose membranes that spreads with time; d) EDS mapping and e) TEM cross‐section of the MXene/cellulose membrane. The magnified picture in the inset of e) shows the layered MXene sheets structure; f) IR thermal pictures of bulk water, and the surface of the membranes of MXene/cellulose, and rGO/cellulose for 0 and 600s; g) SEM image of the MXene/cellulose membrane surface with *E. coli* and S. aureus; h) Densities of S. aureus and *E. coli* on MXene/cellulose, cellulose and rGO/cellulose membranes based on the SEM images; i) Temperatures as a function of irradiation time of the surface of MXene/cellulose, and rGO/cellulose membranes and bulk water under 1 sun solar illumination. Antibacterial efficiencies as a function of contact time for j) *E. coli* and k) S. aureus on MXene/cellulose and rGO/cellulose membranes; l) Pictures of agar plates with the bacterias S. aureus and *E. coli* cells growth exposed to and MXene/cellulose rGO/cellulose, and cellulose, membranes for 24 h contact. Reproduced with permission.^[^ ^237^ ^]^ Copyright 2019, American Chemical Society.

Among the photothermal materials, MXene based materials have been considered as a promising candidate in photothermal membrane fabrication. Very recently, a novel Ti_3_C_2_ MXene on a cellulose membrane (commercially available qualitative cellulose filter paper as depicted in **Figure** **12** ^[^ ^237^ ^]^ with average pore size 30–50 µm was prepared by simply dipping the cellulose filter paper in a MXene solution with a concentration of 1 mg mL^−1^ for 3 s at room temperature and then vacuum dried for 10 min at 60 °C for series of cycles. The as‐prepared membrane exhibited light absorption efficiency as high as ≈94% in a wide solar spectrum range and showed a high antibacterial efficiency up to 99% (**Figure** **13**)^[^ ^237^ ^]^ which was characterized by immersing these in a suspension of *S. aureus* and *E. coli*. The concept design of the MXene/cellulose anti‐biofouling membrane as sunlight capture and the generator of steam for purifying water is shown in Figure 13a. The developed MXene coated cellulose membrane exhibiting a smooth surface (Figure 13c,d) can be folded into different shapes, indicating its flexibility (inset Figure 13b). This work reports the generation of transparent condensed water from the *S. aureus* and *E. coli* bacterial suspensions without any bacterial presence after photothermal water purification (Figure 13l).

### 4.4 MXenes for Removal of Radioactive Wastes

**Figure 14:**
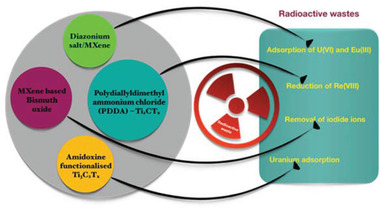
Novel Ti_3_C_2_ MXene for radioactive purposes.^[^ ^242^, ^243^, ^244^ ^]^

**Figure 15:**
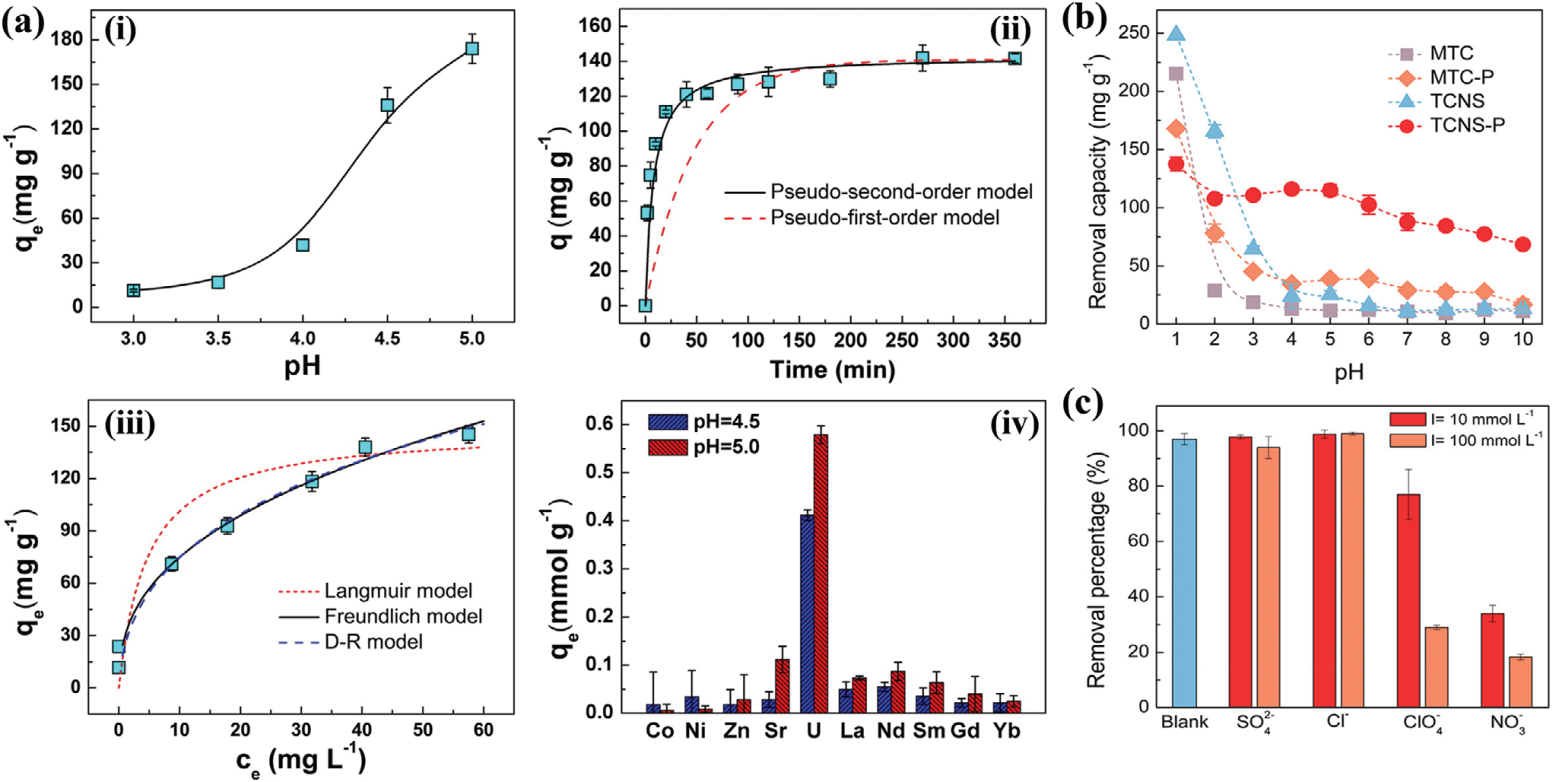
a) U(VI) sorption from aqueous solution onto multi‐layered V2CT*_x_* MXene as a function of i) Ph, ii) contact time, iii) initial U(VI) concentration, and iv) competing metal cations. Reproduced with permission.^[^ ^242^ ^]^ Copyright 2016, American Chemical Society. b) Removal of Anionic Re(VII) from aqueous solution by Ti_2_CT*_x_* MXene and poly(dially/dimethylammonium chloride–PDDA) nanocomposite and c) effect of competitive anion species on Re(VIII) removal by Ti_2_CT*_x_* MXene nanocomposite/PDDA composite. Reproduced with permission.^[^ ^244^ ^]^ Copyright 2019, American Chemical Society.

As nuclear energy is being widely studied and explored, it is important to consider efficient nuclear waste treatment procedures. Although radionuclides are carbon‐free energy sources without carbon emission, their release in considerable amounts may pose significant hazards because of their long half‐lives and high mobility.^[^ ^238^ ^]^ Surface functionalized MXenes have shown an excellent capability in the removal of radioactive isotopes (**Figure** **14**). Several studies have dealt with the removal of uranium using MXenes. Ti_3_C_2_ MXenes were demonstrated in many experiments to adsorb and immobilize radionuclides such as uranium (^235, 238^U),^[^ ^239^ ^]^ cesium (^137^Cs),^[^ ^87^ ^]^ strontium (^90^Sr),^[^ ^237^ ^]^ barium (^133, 140^Ba),^[^ ^238^ ^]^ and thorium (^232^Th)^[^ ^239^, ^240^ ^]^ through straightforward surface.^[^ ^241^ ^]^ This was done by tuning MXenes via intercalation and surface functionalization to encapsulate the radionuclides whose hydrated radii are larger than the layer d‐spacing for MXenes with adsorption capacities.^[^ ^242^, ^243^, ^244^ ^]^ Uranium, the main constituent in nuclear fuel^[^ ^240^, ^245^ ^]^ is a contaminant released into the natural environment during mining and milling processes. Uranium exists as highly mobile uranyl ions which are usually denoted as U(VI).^[^ ^241^, ^246^ ^]^ To explore the adsorptive properties of surface functionalized MXenes for uranium, phenyl carboxylic acid diazonium salt was used to introduce the phenyl carboxylic moiety onto the surface of MXene via a simple synthetic procedure, providing an adsorption capacity for U(VI) of 344.8 mg g^−1^.^[111]^ These hybrid materials also favored the removal of europium, Eu (III), with an adsorption capacity of 97.1 mg g^−1^. It is interesting to note that the surface modification with the phenyl carboxylic moiety increased the stability of the composite material in water. Ling Wang et al. presented a first case study of V_2_CT*_x_* MXene nanosheets with a maximum adsorption capacity uptake of 174 mg g^−1^ (**Figure** **15**a). Density Functional Theory (DFT) study was used to confirm experimental data obtained from X‐Ray absorption showing that uranyl ions prefer to coordinate with hydroxyl groups bonded to the V‐sites of the nanosheets by forming bidentate inner‐sphere complexes.^[^ ^242^ ^]^

Another use of surface functionalized MXenes in the removal of uranium is proposed by Pengcheng and co‐workers by using amidoxime functionalized Ti_3_C_2_T*_x_* MXene.^[^ ^126^ ^]^ They presented kinetic data for U(VI) sorption which showed that more than 95% of uranyl is removed after 5 min at pH 5.0 with the maximum adsorption capacity was 294 mg g^−1^. These same composite materials were loaded with a carbon cloth, where the application of an electric field increased the uranium adsorption capacity from 294 to 626 mg g^−1^.^[^ ^126^ ^]^ The nuclear fission of uranium (^235^U) produces a radioactive element, technetium‐99 (^99^Tc), which has significant demand as a medical radioisotope, is usually present as pertechnetate anion Tc(VII) in an aqueous solution. Wang et al. introduced poly diallyldimethylammonium chloride (PDDA) on to the surface of Ti_3_C_2_T*_x_* MXene nanosheets, which exhibited a removal rate of 363 mg g^−1^ for ReO^−4^; the ReO^−4^ being used as a model chemical surrogate for TcO^−4^ (Tc VII)^[^ ^245^ ^]^ also shows the effect of competitive anion species on Re(VIII) removal by Ti_2_CT*_x_* MXene nanocomposite/PDDA composite (Figure 15b,c). The authors highlighted that the composites were controlled by chemisorption rather than mass transport. By introducing the PDDA, and substituting the existing functional groups leads to an outcome where the electrostatic attraction of Re(VII) by quaternary ammonium cations on the polyelectrolyte PDDA backbone becomes dominant.^[^ ^244^ ^]^

Radioisotopes of iodine have been noted to have a range of half‐lives from ≈8.04 days up to 1.57 × 10^7^ years depending on the isotope. Their sequestration is of high importance given the biological significance of iodine, and the risks posed if its radioisotopes were released/escaped into water bodies. To remove iodide ions, a MXene based bismuth oxide (Mene‐PDA‐Bi_6_O_7_) composite was used. This composite relied on the self‐polymeriyation of dopamine for polydopamine (PDA) films. The maximum adsorption capacity for iodide ions was 64.65 mg g^−1^. It was clear that adsorption was more favorable in acidic solutions than in alkaline solutions. The composite was recycled five times, and the adsorption decreased after the first cycle, because the iodide ions adsorbed were retained by the adsorbent.^[^ ^218^ ^]^ **Table** **2** summarises the various applications of surface functionalized MXenes.

**Table 2:** Summary of various applications of surface functionalized MXenes

Ref	MXene Material	Application	Description/Properties
**Adsorbent**			
^[^ ^198^ ^]^	MXene/cellulose ester filter (MCM)	Removal of methylene blue (MB)	Removal rate of 100% ± 0.1% achieved 90 mg L^−1^ MB concentration
^[^ ^179^ ^]^	Ti_3_C_2_T*_x_*	Removal of copper from water	Adsorption capacity of 78.45 mg g^−1^ (80% of copper adsorbed within 1 min)
^[^ ^178^ ^]^	Alkylated – MXenes (LiOH, KOH, NaOH/Ti_3_C_2_)	Degradation of MB	NaOH modified MXene possessed the highest capacity of 189 mg g^−1 ^(LiOH>KOH)
^[^ ^123^ ^]^	Ti_3_C_2_T*_x_*/PDOPA	Removal of heavy metal ion	Adsorption capacity of heavy metal ions higher with Ti_3_C_2_T*_x_*/PDOPA than with pristine Ti_3_C_2_T*_x_*.
^[^ ^127^ ^]^	Sulfonic groups functionalized Ti_3_C_2_ (Ti_3_C_2_T*_x_*/SO_3_H)	Adsorption of MB	Maximum adsorption capacity of Ti_3_C_2_–SO_3_H was four times that of the raw materials.
^[^ ^101^ ^]^	Ti_3_C_2_T*_x_*/KH570 (silane coupling agent)	Adsorption of lead (Pb^2+^)	Removal rate of 99.99% (147.29 mg g^−1^ at 30 °C) for a 3.2gL^−1^ concentrated solution.
^[^ ^202^ ^]^	Biosurfactant (enzymatic hydrolysis lignin (EHL)/Ti_2_T*_x_*	Adsorption of Pb^2+^	Maximum adsorption capacity of 232.9 mg g^−1^ for Pb(II) ions, resulting in ≈90% removal rate in 10 min.
^[^ ^147^ ^]^	Sodium alginate‐Ti_3_C_2_T*_x_* (MX‐SA)	Adsorption of mercury ions (Hg^2+^)	Exhibited exceptional adsorption of 932.84 mg g^−1^ for Hg^2+^ (100% removal efficiency). Good multi‐component heavy metal ion adsorption, including soft (Hg^2+^ and Cd^2+^), hard (Cr^3+^and As^3+^), and borderline Lewis metal ions (Pb ^2+^, Cu^2+^, Zn^2+^, Ni^2+^, and Co^2+^) and arsenic (As^3+^).
^[^ ^110^ ^]^	Fe_3_O_4_@Ti_3_C_2_T*_x_*‐BA	Adsorption of dopamine and catecholamine	Exhibited high adsorption capacity of up to 319.6 mmol g^−1^ for dopamine and complete adsorption of catecholamines was accomplished within 2.0 min.
^[^ ^136^ ^]^	MoS_2_/MX‐II	Adsorption of mercury ions (Hg^2+^)	Successfully employed in the selective removal of toxic mercuric ions in water and elemental mercury in vapor form.
^[^ ^117^ ^]^	MXene/PEI modified sodium alginate aerogel	Adsorption of Cr(VI) and CR	Exhibited good adsorption capacity of 538.97 mg g^−1^ for Cr(VI) and ultrahigh adsorption capacity of 3568 mg g^−1^ for CR.
^[^ ^106^ ^]^	Imidazoles‐MXene hybrid composite (Ti_3_C_2_@IMIZ	Adsorption of Cr(VI)	Exhibited high adsorption affinity for Cr(VI).
^[^ ^118^ ^]^	Amino‐functionalized MXenes (NH_2_‐Ti_3_C_2_T*_x_*)	Adsorption of Cr(VI)	Possessed strong selective adsorption and reduction ability for Cr(VI) ions in aqueous solution.
^[^ ^189^ ^]^	Functionalized cellulose/MXene composite aerogel (P‐M/MX‐m)	Adsorption of MB	Excellent adsorption performance for MB (168.93 mg g^−1^).
^[^ ^246^ ^]^	Acrylic acid modified alkalized Ti_3_C_2_T*_x_* MXene	Removal of CR and MB	Showed maximum adsorption capacities of 264.46 and 193.92 mg g^−1^, for CR and MB, respectively.
^[^ ^247^ ^]^	Polyimidazole/Ti_3_C_2_T*_x_* MXene (MXene‐PIL)	Adsorption of iodine	Showed a maximum adsorption capacity of 170 mg g^−1^ for iodine and reached an adsorption equilibrium within 10 min.
^[^ ^101^ ^]^	Ionic liquid functionalized MXene (MXene‐PDA‐IL)	Adsorption of iodine	Showed a maximum adsorption capacity of 695.4 mg g^−1^ for iodine.
^[^ ^203^ ^]^	DGO/MXene	Membrane flux	Exhibited an excellent dye rejection ratio 98.1% (for Direct Red 28) and 96.1% (for Direct Black 38) as along with a high value of water flux (63.5 Lm^−2^ h^−1^) at a pressure of 0.1 MPa, compared with the pure MXene and DGO membranes.
^[^ ^116^ ^]^	MXene‐carbon nanotube (CNT)	Membrane flux	Exhibited improved permeability and rejection performance of the membranes. It also possessed potential anti‐swelling property and good stability.
^[^ ^204^ ^]^	PDA@MXene/CA	Membrane flux	Exhibited improved dye separation capability with a rejection ratio of 88.9% for direct red 28, and 88.6% for direct black 38. It also possessed strong anti‐fouling characteristics with good antibacterial ability.
**Photocatalyst**			
^[^ ^152^ ^]^	MXene (Ti_3_C_2_)‐Co_3_O_4_	Degradation of MB and RhB	Removal rate of MB and RB stabilized at ≈240 and 80 min respectively. Material was reused for 8 consecutive cycles.
^[^ ^113^ ^]^	0D/2D Bi_3_TaO_7_/Ti_3_C_2_ heterostructure	Degradation of MB	Degraded 99% MB in 2 h
^[^ ^219^ ^]^	Ag_2_WO4/Ti_3_C_2_	Pharmaceutical wastes	Photocatalytic removal rates of 62.9% for tetracyclinehydrochloride (TC) and 88.6% for sulfadimidine (SFE).
^[^ ^172^ ^]^	g‐C_3_N_4_/Ti_3_C_2_/TiO_2_	Pharmaceutical wastes and organic dyes	Degradation rate constant of RhB and tetracycline hydrochloride by g‐C_3_N_4_/Ti_3_C_2_/TNTAs were about threefold and twofold higher than g‐C_3_N_4_/TNTAs, respectively. g‐C_3_N_4_/Ti_3_C_2_/TNTAs showed good stability
^[^ ^248^ ^]^	2D/2D Ti_3_C_2_/Porous g‐C_3_N_4_	Organic pollutant (phenol)	Ti_3_C_2_/PCN composite showed good day‐photocatalytic capability with 98% phenol removal efficiency. Night‐photocatalysis provided 32% phenol decomposition.
^[^ ^108^ ^]^	Fe_3_O_4_ loaded Ti_3_C_2_	Degradation of MB, RhB, congo red, methyl orange	The Fe_3_O_4_ loaded Ti_3_C_2_ material possessed extremely high degradation efficiency toward RB, MO, CR, and MB (74.82%, 53.07%, 57.29%, and 67.77% in 7, 12, 7, and 6 min, respectively). Further investigations using ESR spectra identified both hydroperoxyl and superoxide radicals as the main species involved in the degradation process.
^[^ ^225^ ^]^	Bi_2_MoO_6_/Ti_3_C_2_	Degradation of tetracycline (TC) and Cr(VI)	Optimized material exhibited the best photocatalytic activity, with removal rates of TC and Cr(VI) both exceeding 99% in a brief time.
^[^ ^163^ ^]^	Bi/BiOCl microspheres on Ti_3_C_2_ nanosheets	Pharmaceutical waste, antibiotic ciprofloxacin (CIP)	Nanocomposite exhibited a higher photocatalytic performance than BiOCl and Bi/BiOCl in degrading CIP under visible light illumination
^[^ ^220^ ^]^	Poly(vinylalcohol)/poly(acrylic acid)/Fe_3_O_4_/MXene@Ag nanoparticle	Reduction of 4‐nitrophenol (4‐NP) & 2‐nitroaniline	Complete degradation recorded after an hour
^[^ ^102^ ^]^	Ti_3_C_2_T*_X_*‐nanosheets/Cu_2_O	Degradation of TC	Degraded 97.6% of TC in 50 min under visible light irradiation
^[^ ^249^ ^]^	Ti_3_C_2_T*_x_* MXene/carbon nanotube (CNTs)	Degradation of RhB	Degraded 75% RhB 3.5 time more than pure RhB
^[^ ^250^ ^]^	ZnO‐Bi_2_WO_6_‐Ti_3_C_2_ MXene	Degradation of CIP	Degraded 77% CIP in 160 min under natural sunlight irradiation
^[^ ^251^ ^]^	Ti_3_C_2_ MXene/WO_3_/PVDF	Degradation of RhB	Maintained a high flux recovery of ≈94% obtained by 5wt% Ti_3_C_2_/WO_3_/PVDF membrane under visible light irradiation after 5 recycling tests
^[^ ^252^ ^]^	Fe_3_O_4_/Ti_3_C_2_T*_x_* MXene	Degradation of MB and RhB	Degraded 91% and 88% MB and RhB, respectively. Adsorption capacities of 153 and 86 mg g^−1^ for MB and RhB, respectively.
^[^ ^253^ ^]^	g‐C_3_N_4_/oxidized Ti_3_C_2_T*_x_*	Reduction of U(VI)	Relatively high extraction capacity of 1615 mg g^−1^ for uranium. Improved reaction constant by 14.05 times fold for the reduction of U(VI) as compared to pristine CN.
^[^ ^254^ ^]^	N‐doped Bi_2_O_2_CO_3_/Ti_3_C_2_T*_x_* MXene	Degradation of CR, trypan blue (TB), and RhB	Degraded 99% of CR, 98% of TB, and 98.4% of RhB. The rejection ratio for three different types of oil/water emulsions was over 99%.
^[^ ^255^ ^]^	Cu_2_O/TiO_2_/Ti_3_C_2_ MXene	Degradation of 2‐nitroaniline (2‐NA) and 4‐nitrophenol (4‐NP)	Exhibited excellent catalysis 2‐NA and 4‐NP with conversion rates greater than 95 and 92% after 8 cycles and with pseudo first‐order reaction rate constants (k) of 0.163 and 0.114 min^−1^, respectively.
**Antibacterial Agents**			
^[^ ^237^ ^]^	MXene/cellulose membrane	Bacteria suspensions of E coli and S aureus	Membrane showed extremely high antibacterial efficiency (above 99.9%) owing to the MXene coating which is a highly effective bacteriostatic agent.
**Radioactive Waste**			
^[^ ^126^ ^]^	Diazonium salt/Mxene	Adsorption of U(VI) and Eu(III)	Optimum adsorption capacities of U(VI) and Eu(III) were 344.8 and 97.1 mg g^−1^, respectively. Diazonium salts increased the stability of the composite material in water.
^[^ ^244^ ^]^	Polydiallyldimethylammonium chloride (PDDA)—Ti_2_CT*_x_*	Reduction of Re(VIII)	Maximum removal capacity of up to 363 mg g^−1^ observed.
^[^ ^218^ ^]^	MXene based Bismuth oxide	Removal of iodide ions	Maximum adsorption capacity of 64.65 mg g^−1^ observed.
^[^ ^243^ ^]^	Amidoxine functionalized Ti_3_C_2_T*_x_*	Uranium adsorption	Composite materials showed adsorption capacity of 294 mg g^−1^. Loading the composite materials with a carbon cloth increased the adsorption capacity to 626 mg g^−1^.

## 5 Summary and Outlook

Rapid progress has been made recently in the realm of wastewater treatment and other applications of MXenes due to their excellent mechanical, thermal, tunable optical, and electronic properties, high ion adsorption abilities, and impressive electric conductivities.^[^ ^45^, ^83^, ^84^ ^]^ In addition to these fascinating properties, the simplistic method of large quantity production of MXene and the possibility of their production in powders to suspensions to thin films make them very attractive to the scientific and industrial community for practical applications. The unique and tunable 2D in‐layer nanostructures and chemical compositions are controllable by functionalizing their surfaces and hence modifying their surface energy. For water treatment, surface functionalized MXenes have shown better results for the adsorption of ionic and polar pollutants and enhanced selectivity in removing heavy metal ions, antimicrobial pollutants, radionuclides, and organic dyes as compared to pristine MXenes. Notably, MXene based materials on different support materials also show significant potential for adsorption of contaminants with water permeability much higher than that of many commercially produced materials. Pristine MXenes have some disadvantages toward photocatalytic applications such as poor photon absorption and a high rate of charge carrier recombination. However, there are plenty of possibilities for MXenes to be used as efficient photocatalysts by changing their surface chemistry. Surface functionalized MXene shows photocatalytic substrates, adsorbents, and antimicrobial agents. Particularly creating an integrated system of MXenes with other support materials can provide cost‐effective nanocomposites with exceptional properties for wastewater treatment, which strongly suggests their potential to be applied in real industrial cases. With improvements on their long‐term stabilities, reductive sequestration and/or adsorption of heavy metals from water and degradation of dyes will be achievable. Although water treatment applications have improved, further development of high‐performing MXene based nanomaterials is very important. MXene‐based nanoarchitectures with higher light‐harvesting ability, satisfying quantum efficiency, and enhanced carrier separation can be used for efficient solar to chemical applications. Plasma processes have been used in modifying other 2D materials to provide improved properties, however, the plasma process has not been fully explored in modifying the surface functionalities of MXenes. Exploring surface functionalized MXenes adsorption interface for other harmful and toxic heavy metal compounds such as Ag, Au, etc, will be an interesting task. Furthermore, increasing the optical absorption range of MXene will make them highly efficient in photocatalytic wastewater treatment, although this remains a challenge.

Overall, MXenes and their composites have rapidly emerged and positioned themself as promising 2D nanomaterial for various applications. Expansion of the family of MXene with the choice of surface functionalization offers new structures, chemistries, and enhanced properties, which still needs to be further explored for sequestration of emerging pollutants from water and wastewater streams. Finally, more research is needed to optimize, enhance, and safely design, use, and dispose of MXene containing products to avoid any risks to the environment.

## Conflict of Interest

The authors declare no conflict of interest.
